# Proteomic and Metabolomic Characteristics of Extremophilic Fungi Under Simulated Mars Conditions

**DOI:** 10.3389/fmicb.2019.01013

**Published:** 2019-05-15

**Authors:** Adriana Blachowicz, Abby J. Chiang, Andreas Elsaesser, Markus Kalkum, Pascale Ehrenfreund, Jason E. Stajich, Tamas Torok, Clay C. C. Wang, Kasthuri Venkateswaran

**Affiliations:** ^1^Department of Pharmacology and Pharmaceutical Sciences, School of Pharmacy, University of Southern California, Los Angeles, CA, United States; ^2^Biotechnology and Planetary Protection Group, Jet Propulsion Laboratory, California Institute of Technology, Pasadena, CA, United States; ^3^Department of Molecular Imaging and Therapy, Beckman Research Institute of City of Hope, Duarte, CA, United States; ^4^Department of Physics, Free University of Berlin, Berlin, Germany; ^5^Leiden Institute of Chemistry, Leiden University, Leiden, Netherlands; ^6^Department of Microbiology and Plant Pathology, Institute of Integrative Genome Biology, University of California, Riverside, Riverside, CA, United States; ^7^Department of Ecology, Lawrence Berkeley National Laboratory, Berkeley, CA, United States; ^8^Department of Chemistry, Dornsife College of Letters, Arts, and Sciences, University of Southern California, Los Angeles, CA, United States

**Keywords:** fungi, simulated mars conditions, proteome, metabolome, Chernobyl, international space station, extremophiles

## Abstract

Filamentous fungi have been associated with extreme habitats, including nuclear power plant accident sites and the International Space Station (ISS). Due to their immense adaptation and phenotypic plasticity capacities, fungi may thrive in what seems like uninhabitable niches. This study is the first report of fungal survival after exposure of monolayers of conidia to simulated Mars conditions (SMC). Conidia of several Chernobyl nuclear accident-associated and ISS-isolated strains were tested for UV-C and SMC sensitivity, which resulted in strain-dependent survival. Strains surviving exposure to SMC for 30 min, ISSFT-021-30 and IMV 00236-30, were further characterized for proteomic, and metabolomic changes. Differential expression of proteins involved in ribosome biogenesis, translation, and carbohydrate metabolic processes was observed. No significant metabolome alterations were revealed. Lastly, ISSFT-021-30 conidia re-exposed to UV-C exhibited enhanced UV-C resistance when compared to the conidia of unexposed ISSFT-021.

## Introduction

Extremophiles are of interest to the National Aeronautics and Space Administration (NASA) due to their potential to survive hostile and extraterrestrial conditions (Rummel et al., 2002). Bacteria, fungi, and archaea have been shown to thrive in habitats characterized by low nutrient availability (Onofri et al., 2004), desiccation (Barnard et al., 2013), high and low temperatures (McKay et al., 2003; Onofri et al., 2007), acidic and alkaline pH (Gonzalez-Toril et al., 2003; Gonsalves, 2012), or radiation (Zhdanova et al., 2004; Dadachova and Casadevall, 2008; Fendrihan et al., 2009; Stan-Lotter and Fendrihan, 2013). There is a need for studies that focus on elucidating microbial survival mechanisms in extreme habitats, as a primary goal of the planetary protection policy is to prevent forward and backward contamination of any celestial bodies and the Earth (Rummel et al., 2002). So far, the majority of such studies have focused on investigating extremophilic bacteria, mainly spore-formers (Nicholson et al., 2012; Tirumalai et al., 2013), and rocks containing fungi (Onofri et al., 2012; Zakharova et al., 2014) however, monolayers of fungal conidia have not been studied.

One investigation revealed that the survival of spacecraft-associated bacteria under simulated Mars UV irradiation, is strain-specific and depends on the isolation site (Newcombe et al., 2005). Spacecraft-associated bacteria were more resistant than *Bacillus subtillis* 168, used as a dosimetric control (Newcombe et al., 2005). Additionally, exposure of *B. subtilis* 168 spores to dark space conditions (no UV) outside the International Space Station (ISS) and SMC for 559 days induced the expression of genes involved in DNA and protein damage responses, and oxidative and envelope stress (Nicholson et al., 2012). Further, the first-generation of spores of another bacterium, *Bacillus pumillus* SAFR-032, isolated from the Jet Propulsion Laboratory (JPL) spacecraft assembly facility (SAF) was more UV-C resistant than the ground control counterparts following exposure to space UV conditions for 18 months. Extensive studies into the whole genome of SAFR-032 revealed the presence of several DNA repair-associated genes that may have facilitated its survival and adaptation to harsh environmental conditions. Further, proteome analysis showed that stress response proteins, like superoxide dismutase, were increased in abundance when compared to the control (Vaishampayan et al., 2012).

Fungi produce conidia or spores as part of their life cycle. Some of these structures can be more resistant to environmental impact than the typical fungal coenocytic cell. Therefore, molecular characterization and further understanding of fungal resistance to UV-C and SMC are of high importance. Additionally, fungi possess a wide variety of mechanisms to protect against solar radiation, which include enzymes removing reactive oxygen species (ROS), DNA repair mechanisms, including nucleotide excision repair (NER) and photoreactivation (PR), and the production of pigments such as melanins and melanin-like compounds, and UV-absorbing metabolites that act as sunscreens (Braga et al., 2015). The hardy nature, adaptability and plasticity of fungi enable their persistence in extreme conditions, making them potential contaminants of cleanrooms and, therefore, a forward contamination source.

On April 25, 1986 one of the most significant nuclear accidents in history took place. Reactor 4 of the Chernobyl nuclear power plant (ChNPP) exploded and high levels of released radioactivity turned the surrounding area into a hostile environment. Within the following 18 years over 2,000 fungal isolates were collected from the nuclear power plant, its 30-km Exclusion Zone and beyond, representing some 200 species in over 90 genera (Zhdanova et al., 2004). The observed fungal communities were of low complexity and dominated by melanin-containing strains. Approximately 20% of the isolates displayed a previously unknown phenomenon of growing toward the radiation source referred to as positive radiotropism (Zhdanova et al., 2004; Dighton et al., 2008). Therefore, this unique characteristic of Chernobyl fungi makes them an ideal fungal model for studying adaptation to SMC. Further, the fungal species isolated from Chernobyl have also been detected in built environments, including JPL clean rooms (La Duc et al., 2012; Weinmaier et al., 2015), simulated closed habitats (Blachowicz et al., 2017), and on board of the ISS (Checinska et al., 2015), which further emphasizes the need to study their potential for forward contamination.

The ISS is another type of environment that may be considered hostile for microorganisms, featuring constant temperature and humidity, controlled airflow, enhanced irradiation, and microgravity (Mora et al., 2016). Several reports examining microbial burden aboard the ISS have shown that fungal populations thrive in this environment (Checinska et al., 2015; Be et al., 2017). Other studies have investigated molecular adaptations of selected species to space conditions, revealing changes in metabolome, and proteome of ISS-isolated strains (Knox et al., 2016; Romsdahl et al., 2018; Blachowicz et al., 2019). In other investigations, several multilayered or embedded cryptoendolithic fungal communities were exposed to space conditions (Billi et al., 2011; Onofri et al., 2012, 2015; Scalzi et al., 2012; Pacelli et al., 2018). Tested isolates that adapted to environmental extremes of their habitats survived SMC for extended periods and revealed high stability of the DNA in the surviving cells (Billi et al., 2011; Onofri et al., 2015; Pacelli et al., 2018). Despite these studies, there are significant gaps in our understanding of the molecular mechanisms that facilitate survival of filamentous fungi under SMC and their potential to adapt and survive in outer space.

This is the first report that evaluated the survival of Chernobyl-associated and ISS-isolated fungal conidia exposed in monolayers to SMC. Dried monolayers of conidia of four filamentous fungi were exposed to SMC and two strains, *Aspergillus fumigatus* and *Cladosporium cladosporoides*, which survived exposure to SMC for 30 min, were further analyzed for phenotypic, proteomic and metabolomic changes.

## Results

### Identification of Fungal Strains

Twelve fungal strains isolated from Chernobyl nuclear accident sites, belonging to nine genera, and one ISS-isolated strain were included in this study. Eight of the Chernobyl isolates were collected over time from the wall surface of the exploded Unit-4 of ChNPP. The other four fungi isolated from soil within the exclusion zone were included for comparison. The strains used in the study were selected based on their ecological and biological significance from the collection of over 2,000 isolates. Speciation was determined by classical cell and colony morphology-based identification techniques. A smaller set of fungi underwent whole genome sequencing. Names, GenBank accession numbers, and their significance are presented in Table 1.

**Table 1:** Fungal isolates used in the study and their significance.

Strain number	Species identification	NCBI accession number	Isolation site	Significance
IMV 00034^∗^	*Cladosporium herbarum*		Wall surface, unit-4, ChNPP	Exhibits radiotropism
IMV 00045^∗^	*Cladosporium sphaerospermum*	MSJI00000000^a^	Wall surface, unit-4, ChNPP	Exhibits radiotropism
IMV 00236^∗^	*Cladosporium cladosporioides*	MSJH00000000^a^	Wall surface, unit-4, ChNPP	Exhibits radiotropism
IMV 00253^∗^	*Acremonium murorum*		Soil, 10-km ChEZ	Exhibits radiotropism
IMV 00265	*Beauveria bassiana*	MSJG00000000^a^	Wall surface, unit-4, ChNPP	Produces tenellin
IMV 00293	*Fusarium oxysporum*	MSJJ00000000^a^	Wall surface, unit-4, ChNPP	Produces dihydronaphthoquinone
IMV 00454	*Trichoderma virens*	MSJK00000000^a^	Soil, 10-km ChEZ	Exhibits auxin-dependent lateral root growth/mycoparasitism
IMV 00738	*Penicillium citreonigrum*		Soil in the ternapol region	European patent 2 333 088
IMV 00882	*Aureobasidium pullulans*	MSJF00000000^a^	Wall surface, unit-4, ChNPP	Produces diketopiperazine
IMV 01167	*Aspergillus terreus*	MSJE00000000^a^	Soil, Kirovograd region	Produces citreoviridin
IMV 01221	*Aspergillus sydowii*		Wall surface, unit-4, ChNPP	Produces sesquiterpeonids
IMV 01851	*Apiospora montagnei*		Wall surface, unit-4, ChNPP	Produces TMC-95A
ISSFT-021	*Aspergillus fumigatus*	KT832787^a^	ISS HEPA filter	Opportunistic pathogen


*ChNPP, Chernobyl nuclear power plant; ChEZ, chernobyl exclusion zone. ^*a*^whole genome sequencing. ^∗^, radiotropism.*

### Survival of Desiccated Conidial Spores Under UV-C Irradiation

**FIGURE 1:**
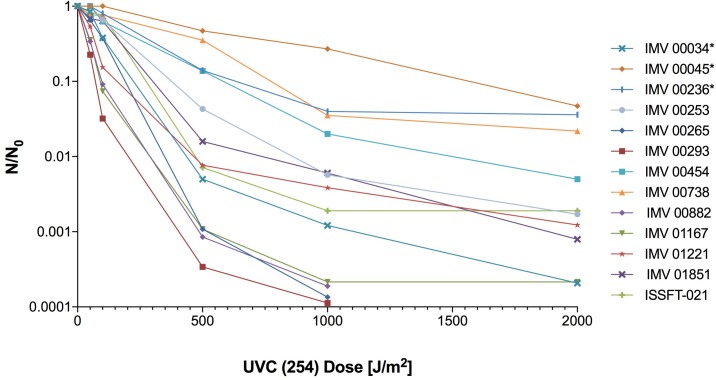
UV-C resistance of Chernobyl and ISS-isolated fungal strains. Purified conidia of 13 strains were exposed to various UV-C doses. The UV-C survival rates were calculated using formula: N/N_0_, # of conidia survived at any given dose/# of conidia exposed at Time 0.

The impact of UV-C irradiation on the survival of dried fungal conidia is presented in Supplementary Table 1. Out of 13 irradiated strains, all but three (*Beauveria bassiana* IMV 00265, *Fusarium oxysporum* IMV 00293, and *Aureobasidium pullulans* IMV 00882) survived exposure to the dose of 2,000 J/m^2^. Two radiotropic strains, *Cladosporium sphaerospermum* IMV 00045, and *Cladosporium cladosporioides* IMV 00236, and the non-radiotropic *Penicillium citreonigrum* IMV 00738 showed survival at the level of 3.48, 3.60, and 2.18%, respectively, which was higher than the ∼0.1% survival rate observed for the other strains (Figure 1 and Supplementary Table 1). Rapid decrease in conidia survivability was observed at dose of 500 J/m^2^ (∼2–3 log reduction). From that point until the doses of 1000 or 2000 J/m^2^, the decrease in survival was less pronounced.

### Survival of Desiccated Conidia Under Simulated Mars Conditions (SMC)

Based on the initial analysis of survival under SMC (not shown) four fungal strains were exposed to SMC for 5 and 30 min at the Leiden Institute of Chemistry, Netherlands. All exposed strains, *C. cladosporioides* IMV 00236, *Apiospora montagnei* IMV 01851, *C. herbarum* IMV 00034 and *A. fumigatus* ISSFT-021, survived exposure to SMC for 5 min, whereas only two strains: *C. cladosporioides* IMV 00236 and *A. fumigatus* ISSFT-021 survived exposure for 30 min (Table 2). Probably the most striking observation was the highest survival rate of IMV 00034 after 5 min exposure to SMC followed by complete eradication after 30 min exposure to SMC. However, because the main focus of the manuscript was to discuss the potential of fungi to survive long-term exposure to SMC we did not further investigate why IMV 00034 may be highly tolerant to SMC-exposure for the short time, but it fails to survive during longer exposure. Nevertheless, it remains an interesting question and a topic for further investigation.

**Table 2:** Quantitative analysis of the simulated Mars conditions (SMC) tolerance of selected extremotolerant Chernobyl- and ISS-isolated fungi.

	Growth after exposure of cultures to simulated Mars conditions (SMC)^b^				
**Strain^a^**	**Unexposed**	**SMC – 5 min**	**SMC – 30 min**	**Percent of survival – 5 min exposure**	**Percent of survival – 30 min exposure**
IMV 00034^∗^	+	+	-	21.11	0.00
IMV 00236^∗^	+	+	+	4.14	2.83
IMV 01851	+	+	-	4.17	0.00
ISSFT-021	+	+	+	15.00	2.50


*^*a*^IMV, Institute for Microbiology and Virology (academy of sciences), Kiyv, Ukraine. ISSFT, international space station filter; ^∗^, radiotropism. ^*b*^“+” growth after exposure. “-”, no growth after exposure.*

### Secondary Metabolite (SM) Profiling of *Aspergillus fumigatus* and *Cladosporium cladosporioides* Exposed to SMC

**FIGURE 2:**
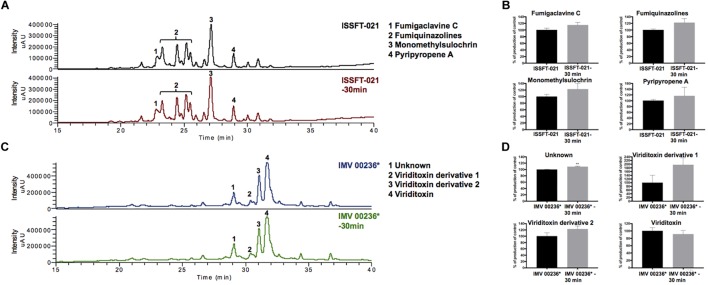
Secondary metabolite production of SMC-exposed ISSFT-021-30 and IMV 00236-30 when compared to unexposed ISSFT-021 and IMV 00236. **(A)** Secondary metabolite profiles of ISSFT-021-30 and ISSFT-021 when grown on GMM. **(B)** Secondary metabolite profiles of IMV 00236-30 and IMV 00236 when grown on MEA. **(C)** Metabolite quantification, showing the percent change for each metabolite in relation to unexposed ISSFT-021; significance was determined using Welch’s *t*-test. **(D)** Metabolite quantification, showing the percent change for each metabolite in relation to unexposed IMV 00236; significance was determined using Welch’s *t*-test. ^∗^, radiotropism.

Organic extracts of unexposed ISSFT-021 and IMV 00236, and 30 min SMC-exposed ISSFT-021-30 and IMV 00236-30 strains were examined to test if exposure to SMC alters SM production. No significant differences were observed in SM production when comparing samples before and after SMC exposure, including yield or type of compound produced, in either of the strains (Figure 2). However, there appeared to be a tendency of increased SM production yield in both strains following exposure to SMC when compared to unexposed counterparts (Figure 2B,D).

### Proteome Profiling of *Aspergillus fumigatus* Exposed to SMC

**FIGURE 3:**
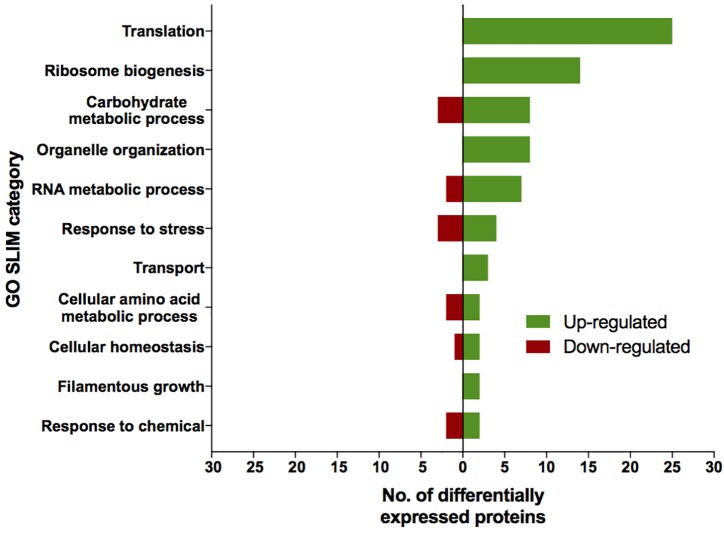
AspGD GO Slim terms of differentially expressed proteins in ISSFT-021-30. Differentially expressed proteins in (FC > |2|, *P* < 0.05) were mapped to terms representing various biological processes using AspGD gene ontology (GO) Slim Mapper.

The proteomic characterization of SMC-exposed ISSFT-021-30 revealed 51-up and 24 down-regulated proteins when compared to unexposed ISSFT-021 (fold-change (FC) > | 2|, *P* < 0.05) (Supplementary Table 2). Analysis of the distribution of differentially expressed proteins among biological processes revealed that 27 proteins were involved in translation and ribosome biogenesis, 11 in carbohydrate metabolism, and 10 in stress response (Figure 3). Further, significantly over-represented up-regulated biological processes included translation (50% of all up-regulated proteins), and carbohydrate metabolic processes (15%), whereas significantly over-represented down-regulated processes included carbohydrate derivative metabolic processes (12%) (Supplementary Table 3).

Approximately 50% of all up-regulated proteins in ISSFT-021-30 were involved in translation and ribosome biogenesis, including proteins that comprise the small and large ribosomal subunit (Table 3). The majority of these proteins were reported to be differentially expressed during the early development of *A. fumigatus* (Cagas et al., 2011). Both Rpl3 (AFUA_2G11850) and ribosomal protein P0 (AFUA_1G05080), whose expression during conidiation are regulated by BrlAp, were more than twofold up-regulated (Twumasi-Boateng et al., 2009). A number of differentially expressed proteins were involved in carbohydrate metabolism (Table 4), including isocitrate lyase AcuD (AFUA_4G13510), one of the key enzymes in the glyoxylate cycle (Olivas et al., 2008). Cellobiohydrolases CbhB (AFUA_6G11610) and AFUA_8G01490, both involved in cellulose degradation, and hydrolase AFUA_5G07080 were more than twofold up-regulated in SMC-exposed ISSFT-021-30 (Adav et al., 2013). A twofold increase in protein abundance was observed for glycerol dehydrogenase GldB (AFUA_4G11730) (Teutschbein et al., 2010), phosphoglycerate kinase (AFUA_1G10350), and hexokinase HxkA (AFUA_2G05910) (Albrecht et al., 2010). Among the proteins involved in carbohydrate metabolism that exhibited decreased abundance were malate and alcohol dehydrogenases [AFUA_6G05210 (Teutschbein et al., 2010), AFUA_5G06240 (Cagas et al., 2011), respectively] and mannose-6-phosphate isomerase (AFUA_4G08410) (Gautam et al., 2011). Several proteins with increased abundance were involved in response to stress (Table 5). Dehydrin-like protein DprC (AFUA_7G04520), which is known to play a role in protecting cells against freezing (Hoi et al., 2011), and AFUA_1G14090, which is predicted to be involved in histidine biosynthesis (Nierman et al., 2005), were twofold up-regulated. Down-regulated stress response proteins included the proliferating cell nuclear antigen (PCNA) (AFUA_1G04900), and the formaldehyde dehydrogenase (AFUA_2G01040), and AFUA_8G04890 with a predicted role in response to salt stress (Nierman et al., 2005).

**Table 3:** Differentially expressed proteins involved in translation and ribosome biogenesis in ISSFT-021-30 subjected to SMC.

ORF	Protein	Relative protein abundance^∗^	*P*-value	Putative function/activity
AFUA_5G05630		2.15	1.19E-03	60S ribosomal protein L23
AFUA_6G05200		2.01	4.67E-02	60S ribosomal protein L28
AFUA_4G03880		1.66	7.86E-03	60S ribosomal protein L7
AFUA_4G07435		1.60	2.68E-04	60S ribosomal protein L36
AFUA_5G06360		1.59	1.46E-03	60S ribosomal protein L8
AFUA_2G03380		1.58	2.37E-03	large ribosomal subunit
AFUA_4G07730		1.56	3.32E-02	60S ribosomal protein L11
AFUA_1G03390		1.54	4.06E-03	60S ribosomal protein L12
AFUA_1G09100		1.47	1.38E-03	60S ribosomal protein L9
AFUA_6G11260		1.46	6.21E-03	Ribosomal protein L26
AFUA_5G03020		1.43	3.84E-02	60S ribosomal protein L4
AFUA_2G11850	Rpl3	1.39	2.36E-04	Allergenic ribosomal L3 protein
AFUA_2G16370		1.39	4.93E-03	60S ribosomal protein L32
AFUA_1G14410	Rpl17	1.36	5.82E-03	60S ribosomal protein L17
AFUA_2G09210		1.34	1.17E-02	60S ribosomal protein L10
AFUA_2G03040		1.34	4.84E-02	Ribosomal protein L34
AFUA_3G06760		1.32	1.48E-02	Ribosomal protein L37
AFUA_1G05080		1.27	5.66E-03	60S ribosomal protein P0
AFUA_4G04460		1.25	5.15E-03	60S ribosomal protein L13
AFUA_3G13480		1.22	3.30E-02	Translation initiation factor 2 alpha subunit
AFUA_6G03830		1.22	3.22E-03	Ribosomal protein L14
AFUA_6G12660		1.18	2.46E-02	40S ribosomal protein S10b
AFUA_1G11130		1.18	2.20E-02	60S ribosomal protein L6
AFUA_1G12890		1.17	1.89E-03	60S ribosomal protein L5
AFUA_2G09200		1.10	1.83E-03	60S ribosomal protein L30
AFUA_2G16010		1.07	2.97E-02	Prolyl-tRNA synthetase
AFUA_2G03590	Rps21	1.05	8.30E-04	Ribosomal protein S21e


*^∗^Log2 fold change of ISSFT-021-30 compared to ISSFT-021 (*P* < 0.05).*

**Table 4:** Differentially expressed proteins involved in carbohydrate metabolism in ISSFT-021-30 subjected to SMC.

ORF	Protein	Relative protein abundance^∗^	*P*-value	Putative function/activity
AFUA_4G13510	AcuD/Icl1	1.78	4.60E-03	Isocitrate lyase involved in the glyoxylate cycle
AFUA_6G11610	CbhB	1.42	3.85E-03	Cellobiohydrolase
AFUA_5G07080		1.39	4.37E-02	Hydrolase
AFUA_8G01490		1.32	1.64E-02	Cellobiohydrolase
AFUA_4G11730	GldB	1.08	3.70E-02	Glycerol dehydrogenase
AFUA_1G10350		1.03	4.74E-03	Phosphoglycerate kinase
AFUA_2G05910	HxkA	1.01	1.07E-02	Hexokinase
AFUA_1G06960		1.00	3.76E-02	Pyruvate dehydrogenase complex subunit alpha
AFUA_6G05210		-1.18	1.36E-04	Malate dehydrogenase involved in the citric acid cycle
AFUA_5G06240	AlcC	-1.22	1.83E-04	Alcohol dehydrogenase
AFUA_4G08410		-2.23	1.53E-02	Mannose-6-phosphate isomerase


*^∗^Log2 fold change of ISSFT-021-30 compared to ISSFT-021 (*P* < 0.05).*

**Table 5:** Differentially expressed proteins involved in response to stress in ISSFT-021-30 subjected to SMC.

ORF	Protein	Relative protein abundance^∗^	*P*-value		Putative function/activity
AFUA_1G14410	Rpl17	1.36	5.82E-03	60S ribosomal protein L17	
AFUA_3G13480		1.22	3.30E-02	Translation initiation factor 2 alpha subunit	
AFUA_7G04520	DprC	1.21	5.74E-03	Dehydrin-like protein, acts downstream of SakA to confer cold tolerance	
AFUA_1G11130		1.18	2.20E-02	60S ribosomal protein L6	
AFUA_1G14090		1.15	5.56E-03	Histidine biosynthesis	
AFUA_4G11730	GldB	1.08	3.70E-02	Glycerol dehydrogenase	
AFUA_8G04890		-1.21	4.06E-02	Role in response to salt stress	
AFUA_1G15450		-1.31	4.62E-03	Adenylosuccinate synthase	
AFUA_1G04900		-1.57	4.26E-03	Proliferating cell nuclear antigen (PCNA)	
AFUA_2G01040		-2.67	8.06E-04	Formaldehyde dehydrogenase	


*^∗^Log2 fold change of ISSFT-021-30 compared to ISSFT-021 (*P* < 0.05).*

### Proteome Profiling of *Cladosporium cladosporioides* Exposed to SMC

**FIGURE 4:**
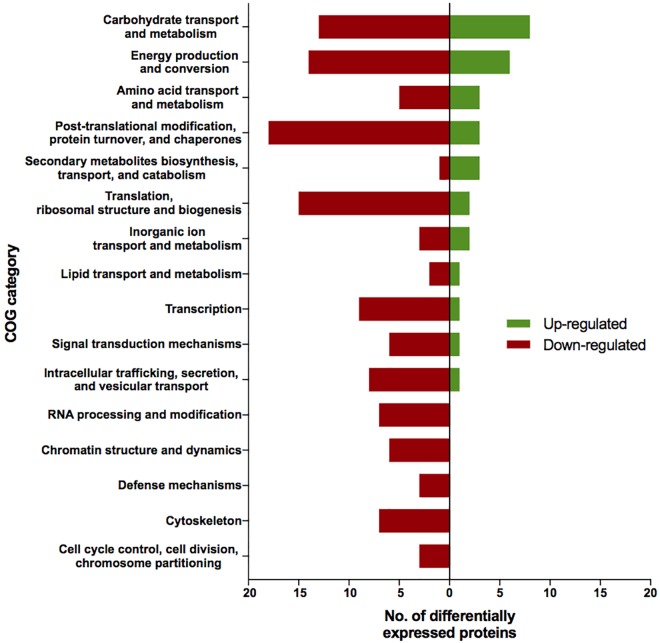
Biological process COG categories of differentially expressed proteins in IMV 00236-30. Differentially abundant proteins (FC > |2|, *P* < 0.05) were mapped to terms representing various biological processes using cluster of orthologous genes (COG) database in CloVR.

The proteomic characterization of *C. cladosporioides* upon exposure to SMC for 30 min revealed that 51 proteins were up-regulated, and 218 proteins were down-regulated when compared to unexposed IMV 00236 (fold-change (FC) > ∣2∣, *P* < 0.05) in response to SMC (Supplementary Table 4). The distribution of differentially expressed proteins in SMC-exposed IMV 00236-30 among biological processes is shown in Figure 4. Among differentially expressed proteins 22 were involved in post-translation modification, protein turnover, and chaperones, 21 in carbohydrate transport and metabolism, 20 in energy production and conversion, and 17 in translation and ribosomal structure and biogenesis. Interestingly, the majority of the proteins involved in the translation and ribosomal structure and biogenesis in SMC-exposed IMV 00236-30 exhibited down-regulation (Table 6), which is the opposite expression pattern to ISSFT-021-30. Additionally, a number of proteins involved in post-translational modification and chaperones (Table 7) showed decreased abundance, including an aspartic endopeptidase (BS090_008183/ENOG410PH8I), which is an ortholog of *A. fumigatus* AFUA_5G13300 (Nierman et al., 2005). BS090_010805/ENOG410PMR5, an ortholog of mitochondrial matrix cochaperone Mge1p (YOR232W) in *Saccharomyces cerevisiae* (Costanzo et al., 2010) was fourfold down-regulated when compared to SMC-unexposed IMV 00236. Proteins involved in carbohydrate metabolism displayed differential abundance (Table 8), including the fourfold up-regulated exo-polygalacturonase involved in pectin degradation BS090_001871/ENOG410PG7M, which is an ortholog of An12g07500 in *Aspergillus niger* (Martens-Uzunova et al., 2006). Chitin deacetylases BS090_000013 and BS090_000044/ENOG410PMJX, and the chitin recognition protein BS090_010953/ENOG410PMF7 were at least threefold up-regulated. Down-regulated proteins involved in carbohydrate metabolism included phosphoglycerate mutase BS090_004087/ENOG410QEDC, which is an ortholog of *Aspergillus nidulans* AN8720 with a predicted role in gluconeogenesis and glycolysis (Masuo et al., 2010), glucanase BS090_003291/ENOG410PM6H, and alpha-amylase BS090_011829/ENOG410PMDW. Differentially expressed proteins involved in energy production and conversion (Table 9) included up-regulated BS090_001715/ENOG410PGTG, an ortholog of *A. niger* NADPH dehydrogenase (An11g08510), and isocytrate lyase and dehydrogenase BS090_001881/ENOG410PGND, and BS090_000939/ENOG410PFHR, respectively. Proteins with decreased abundance included nitrate reductase BS090_004112/ENOG410PUCE and ATP synthase BS090_005644/ENOG41KOG1758.

**Table 6:** Differentially expressed proteins involved in translation, ribosomal structure, and biogenesis in IMV 00236-30 subjected to SMC.

Accession	Relative protein abundance^∗^	*P*-value	EggNog	EggNog annotation
BS090_000406	1.44	2.67E-03	ENOG410PN3M	40S ribosomal protein S12
BS090_001853	1.13	1.89E-03	ENOG410PG8Y	Ribosomal protein L15
BS090_003273	-1.03	1.19E-02	ENOG410PN86	La domain
BS090_000015	-1.12	3.60E-03	ENOG410PMV6	40s ribosomal protein S17
BS090_011340	-1.16	5.27E-03	ENOG410PNPS	Translation initiation factor
BS090_009099	-1.18	3.88E-02	ENOG410PGC2	Eukaryotic translation initiation factor 5
BS090_002173	-1.23	7.84E-03	ENOG410PP8V	60S ribosomal protein L31
BS090_005915	-1.24	6.15E-03	ENOG410PHV2	Prolyl-tRNA synthetase
BS090_006863	-1.35	2.19E-04	ENOG410PPAS	60s ribosomal protein
BS090_002466	-1.35	3.65E-03	ENOG410PQS0	Processing of the 20S rRNA-precursor to mature 18S rRNA
BS090_003537	-1.35	7.24E-03	ENOG410PI34	Component of the eukaryotic translation initiation factor 3 (eIF-3) complex
BS090_000480	-1.48	1.36E-02	ENOG410PFEB	Seryl-tRNA synthetase
BS090_001464	-1.53	1.78E-03	ENOG410PRUB	60S acidic ribosomal protein P2
BS090_006330	-1.65	3.30E-03	ENOG410PQ50	Ribosome biogenesis protein Nhp2
BS090_009967	-1.81	1.19E-02	ENOG410PRWG	60S acidic ribosomal protein P1
BS090_006228	-2.13	5.69E-03	ENOG410PP4P	L-PSP endoribonuclease family protein (Hmf1)
BS090_007862	-2.52	1.56E-03	ENOG410PQSK	60S ribosomal protein L22


*^∗^Log2 fold change of IMV 00236-30 compared to IMV 00236 (*P* < 0.05).*

**Table 7:** Differentially expressed proteins involved in post-translational modification, protein turnover, and chaperones in IMV 00236-30 subjected to SMC.

Accession	Relative protein abundance^∗^	*P*-value	EggNog	EggNog annotation
BS090_010341	1.55	5.80E-03	ENOG410PJAB	Thioredoxin reductase
BS090_002416	1.42	3.68E-03	ENOG41KOG1339	Aspartic
BS090_010922	1.12	3.34E-03	ENOG410PX4S	OsmC-like protein
BS090_011023	-1.01	1.52E-03	ENOG410PNQ9	Peptidyl prolyl cis-trans isomerase Cyclophilin
BS090_008028	-1.12	4.88E-02	ENOG410PPYQ	Ubiquitin conjugating enzyme
BS090_005834	-1.20	6.64E-04	ENOG410PJ50	26S proteasome non-ATPase regulatory subunit 11
BS090_010452	-1.24	1.83E-02	ENOG410PQY3	Peptidyl-prolyl cis-trans isomerase
BS090_010972	-1.28	3.67E-03	ENOG410PP80	Cupin domain protein
BS090_011316	-1.32	7.31E-03	ENOG410PKHZ	Protein-L-isoaspartate O-methyltransferase
BS090_005149	-1.33	2.63E-03	ENOG410PP3T	Subunit 3
BS090_009030	-1.34	1.91E-03	ENOG410PP19	Peptidyl-prolyl cis-trans isomerase
BS090_004399	-1.59	1.87E-03	ENOG410PPJH	Heat shock protein
BS090_007304	-1.63	2.98E-03	ENOG410PHFF	Protease S8 tripeptidyl peptidase I
BS090_009384	-1.63	1.01E-03	ENOG410PI5I	Tripeptidyl-peptidase
BS090_008147	-1.81	3.06E-03	ENOG41KOG0541	Peroxiredoxin
BS090_005718	-1.90	1.72E-04	ENOG410PGPE	Disulfide-isomerase
BS090_010805	-2.03	1.03E-02	ENOG410PMR5	Component of the PAM complex
BS090_010009	-2.07	3.52E-02	ENOG410PQV2	Prefoldin subunit 6
BS090_008183	-2.19	1.69E-03	ENOG410PH8I	Aspartic endopeptidase
BS090_009852	-2.47	6.17E-03	ENOG410PSDM	Glutaredoxin
BS090_008141	-3.13	6.96E-03	ENOG410PRTR	Heat shock protein


*^∗^Log2 fold change of IMV 00236-30 compared to IMV 00236 (*P* < 0.05).*

**Table 8:** Differentially expressed proteins involved in carbohydrate transport and metabolism in IMV 00236-30 subjected to SMC.

Accession	Relative protein abundance^∗^	*P*-value	EggNog	EggNog annotation
BS090_010953	2.31	8.94E-04	ENOG410PMF7	Chitin recognition protein
BS090_001871	2.14	3.38E-03	ENOG410PG7M	Exo-polygalacturonase
BS090_000013	1.95	1.89E-02	ENOG410PMJX	Chitin deacetylase-like mannoprotein MP98
BS090_003824	1.93	9.34E-03	ENOG410PMRY	LysM domain
BS090_001404	1.67	6.92E-03	ENOG41KOG1458	Fructose-1,6-bisphosphatase
BS090_000044	1.58	8.22E-03	ENOG410PMJX	Chitin deacetylase-like mannoprotein MP98
BS090_000502	1.28	7.07E-03	ENOG410PJKF	Catalyzes the epimerization of the S- and R-forms of NAD(P)HX
BS090_011896	1.21	9.04E-03	ENOG410PK51	Glyco_18
BS090_010859	-1.09	2.80E-02	ENOG410PGWP	Mannose-6-phosphate isomerase
BS090_011008	-1.10	5.27E-04	ENOG410PG84	Beta-glucosidase
BS090_007695	-1.13	2.36E-03	ENOG410PJ6P	Glucan 1,4-alpha-glucosidase
BS090_008700	-1.16	5.40E-03	ENOG410PK8I	WSC domain
BS090_006133	-1.17	5.18E-03	ENOG410PF9K	Glyceraldehyde-3-phosphate dehydrogenase
BS090_002903	-1.37	3.43E-02	ENOG410PJIN	Glycolipid transfer protein HET-C2
BS090_003039	-1.47	9.13E-04	ENOG410PH6W	Cell wall
BS090_008425	-1.49	2.12E-03	ENOG410PIQS	Snf1 kinase complex beta-subunit Gal83
BS090_003291	-1.51	4.22E-03	ENOG410PM6H	Glucanase
BS090_011829	-1.66	1.43E-03	ENOG410PMDW	Alpha-amylase
BS090_011280	-1.91	8.40E-03	ENOG410PKN9	Oxalate decarboxylase
BS090_004931	-2.02	3.61E-02	ENOG410PM9J	Major intrinsic protein
BS090_004087	-2.34	9.93E-05	ENOG410QEDC	Phosphoglycerate mutase


*^∗^Log2 fold change of IMV 00236-30 compared to IMV 00236 (*P* < 0.05).*

**Table 9:** Differentially expressed proteins involved in energy production and conversion in IMV 00236-30 subjected to SMC.

Accession	Relative protein abundance^∗^	*P*-value	EggNog	EggNog annotation
BS090_001053	1.45	2.24E-03	ENOG410PGVS	Oxidoreductase
BS090_001715	1.25	3.00E-03	ENOG410PGTG	NADH flavin oxidoreductase NADH oxidase family protein
BS090_001881	1.23	8.71E-03	ENOG410PGND	Isocitrate lyase
BS090_000071	1.16	1.95E-03	ENOG410PFIA	Component of the ubiquinol-cytochrome c reductase complex
BS090_007434	1.04	1.25E-03	ENOG410PI78	Phosphoenolpyruvate carboxykinase
BS090_000939	1.03	1.87E-02	ENOG410PFHR	Isocitrate dehydrogenase NADP
BS090_000792	-1.02	1.27E-02	ENOG410PN6K	Mitochondrial membrane ATP synthase [F(1)F(0) ATP synthase or complex V]
BS090_004086	-1.11	4.06E-02	ENOG410PFM5	Inorganic pyrophosphatase
BS090_011745	-1.14	4.04E-02	ENOG410PNH4	Conserved hypothetical protein
BS090_003155	-1.19	5.65E-03	ENOG410PNBT	Regulatory protein SUAPRGA1
BS090_010935	-1.23	1.25E-02	ENOG410PFFW	Electron transfer flavoprotein
BS090_011347	-1.26	1.29E-03	ENOG410PNPT	Cytochrome c oxidase polypeptide VIa
BS090_003274	-1.27	8.98E-05	ENOG410PFBB	Stomatin family
BS090_005666	-1.28	4.59E-03	ENOG410PH2F	Mitochondrial membrane ATP synthase [F(1)F(0) ATP synthase or Complex V]
BS090_006999	-1.33	2.00E-03	ENOG410PFI6	Electron transfer flavoprotein
BS090_004112	-1.51	1.16E-02	ENOG410PUCE	Nitrate reductase
BS090_011473	-1.53	5.63E-03	ENOG410PJA9	Vacuolar ATP synthase subunit e
BS090_008223	-1.59	8.26E-03	ENOG410PNQY	Iron sulfur cluster assembly protein
BS090_008509	-2.04	2.62E-03	ENOG410PS16	Mitochondrial ATP synthase epsilon chain domain-containing protein
BS090_005644	-2.40	4.70E-03	ENOG41KOG1758	ATP synthase


*^∗^Log2 fold change of IMV 00236-30 compared to IMV 00236 (*P* < 0.05).*

### Increased Resistance to UV-C of SMC Exposed *Aspergillus fumigatus* Conidia

**FIGURE 5:**
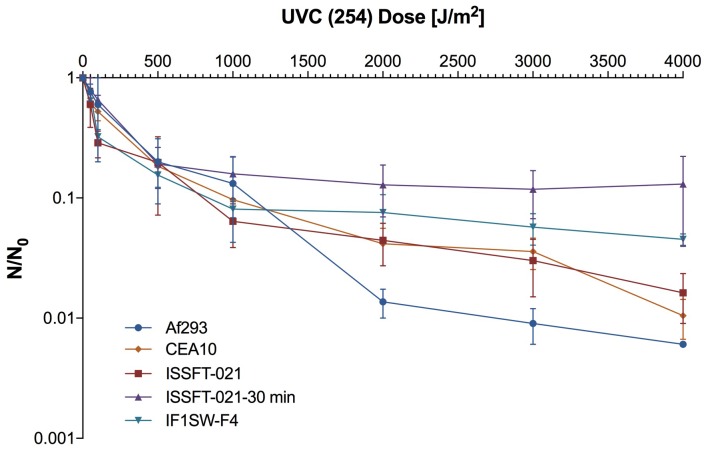
UV-C resistance of *A. fumigatus* ISS-isolated and clinical strains. Purified conidia of ISS-isolated (ISSFT-021 and IF1SW-F4) SMC-exposed (ISSFT-021-30), and clinical isolates (Af293 and CEA10) were exposed to varying doses of UV-C. The UV-C survival rates were calculated using formula: N/N_0_, # of conidia survived at any given dose/# of conidia exposed at Time 0. The average fungal conidia survival rates from three different experiments are plotted.

Survival rates of several ISS-isolated and clinical isolates of *A. fumigatus* following exposure to UV-C are presented in Figure 5. SMC exposed ISSFT-021-30 exhibited increased UV-C resistance (∼20% of conidia survived the UV-C dose of 4,000 J/m^2^) when compared to unexposed ISSFT-021 and another *A. fumigatus* ISS isolate IF1SW-F4. Additionally, IF1SW-F4 was more resistant to UV-C exposure, than ISSFT-021 and clinical CEA10 strain, which displayed similar resistance patterns. The clinical isolate Af293 was the most sensitive, showing 2-log reduction when exposed to the highest tested UV-C dose. Exposure experiments were repeated 3 times and showed the same trends.

## Discussion

Although it has been well documented that bacteria are associated with the spacecraft environment (La Duc et al., 2003, 2004; Newcombe et al., 2005), few studies address the persistence of fungi in this environment (La Duc et al., 2012; Weinmaier et al., 2015). Prepared in monolayers, spacecraft-associated spore-forming bacteria have been shown to survive exposure to UV-C and SMC (Newcombe et al., 2005; Osman et al., 2008), but similar studies have not been performed for fungal conidia. Microorganisms exposed as multilayers are shielded from UV penetration (submicron level) and subsequent UV-damage is prevented, therefore generation of monolayers of tested cells/spores/conidia is crucial for characterizing irradiation-induced microbial lethality. The observed strain-dependent UV-C sensitivity of fungal conidia was not surprising, as similar conclusions were drawn for UV-exposed bacterial species (Osman et al., 2008). Interestingly, ten out of thirteen selected fungal species survived the UV-C dose of 2,000 J/m^2^, while bacterial species in a similar study survived exposure to 1,000 J/m^2^ (Osman et al., 2008), suggesting that some extremophilic fungi are as hardy, if not more, than tested bacteria. It should be noted, that conducting experiments that investigate the protective nature of fungi or bacteria could help in understanding the microbial survival in extreme niches, as bacteria and fungi coexist in the environment, and form communities to survive harsh conditions.

The space environment varies significantly from Earth. It is characterized by enhanced irradiation and distinct atmospheric conditions. Therefore, it was imperative to assess fungal survival under SMC, as fungi are known to be present during manned space missions. Among the four strains that survived 5-min exposure to SMC two, ISSFT-021 and IMV 00236, survived exposure to SMC for 30 min. Most studies testing survival of microorganisms under SMC have been conducted using bacterial spores or fungal communities. In one such study several *Bacillus* spp. were tested for simulated Mars UV irradiation tolerance, which resulted in no survival beyond 30 min exposure for all strains except *B. pumilus* SAFR-032, which was inactivated after 180 min of exposure (Schuerger et al., 2006). However, when *B. subtilis* spores were exposed to SMC, including irradiation and atmospheric conditions, 99.9% of spores were eradicated within 30 s, and a 15 min exposure resulted in no recovery of viable spores from aluminum coupons (Schuerger et al., 2003). Interestingly, when the more extremotolerant *B. pumilus* SAFR-032 was tested under SMC, no spores were recovered from the aluminum coupon after 30 min exposure, but bacterial growth was observed once coupons were placed in tryptic soy broth (TSB) (Osman et al., 2008). In this study, recovery of fungal conidia exposed in monolayers from aluminum coupons was possible for both ISSFT-021 and IMV 00236 even after 30 min exposure, suggesting an enhanced ability of fungal conidia to withstand such environments. The results from this study, combined with those revealing that cryptoendolithic fungal communities embedded in rocks can withstand SMC for an extended period of time (Onofri et al., 2012, 2015; Pacelli et al., 2018), imply that fungi should be considered as a possible forward contamination source. This is further supported by the fact that the omnipresence of filamentous fungi has been documented in spacecraft assembly facilities (La Duc et al., 2012; Weinmaier et al., 2015).

It has been reported that upon exposure to space conditions bacteria become more UV resistant (Vaishampayan et al., 2012). Similarly, this study showed that SMC-exposed ISSFT-021-30 had a higher tolerance to UV irradiation than its unexposed counterpart ISSFT-021. Additionally, it appeared to be more tolerant to UV exposure than any of the additionally tested *A. fumigaus* strain, including another ISS-isolated strain IF1SW-F4 and two clinical isolates Af293 and CEA10. These results suggest that exposure to an enhanced irradiation environment may lead to adaptive alterations that give fungi an increased environmental advantage when exposed to unique space conditions.

One significant way that filamentous fungi respond to external stimuli is through alterations in SM production. Although these bioactive molecules are not directly necessary for survival, they often confer an environmental advantage (Keller et al., 2005). Both fungal species subjected to SMC, ISSFT-021-30 and IMV 00236-30, displayed slightly increased yield of produced SMs. Such a tendency agrees with previously observed elevated yields of SM in ISS-isolated JSC-093350089 *A. niger* when compared to culture collection strain ATCC 1015 (Romsdahl et al., unpublished). Additionally, when the metabolome of ISSFT-021 was characterized and compared to the well-studied Af293, production yields of pyripyropene A and fumiquinazolines increased (Knox et al., 2016). Therefore, the observed tendency of increased SM production in strains following exposure to SMC supports the hypothesis that space conditions might have altered secondary metabolite production yields.

This study revealed that exposure to SMC altered the proteome of both ISSFT-021-30 and IMV 00236-30 when compared to unexposed counterparts. Interestingly, in both species, the highest number of differentially expressed proteins were translation-related ribosomal components. Interestingly, exposed to ionizing radiation *C. sphaerospermum, Wangiella dermatitidis*, and *Cryptococcus neoformans* showed increased growth when compared to unexposed controls due to electronic changes in melanin (Dadachova et al., 2007), however, in our study only ISSFT-021-30 seemed to follow that pattern, revealing up-regulation of translation-related proteins. Observed opposite expression patterns of translation-related proteins, which underlay species-related unique defense system, may have been shaped by the varying environmental origins of each isolate (Gasch, 2007; Heitman et al., 2017). This discrepancy suggests that different species of filamentous fungi alter their growth and development in response to adverse environmental conditions in a species/strain-specific manner. Furthermore, the difference in the expression levels of translation-related ribosomal protein may lead to the overall up- and down-regulation of other proteins in ISSFT-021-30 and IMV 00236-30, respectively. Interestingly, ribosomal protein Rpl17, has been indicated as crucial for survival in *A. fumigatus* (Firon et al., 2003), *C. neoformans* (Ianiri et al., 2017), and *S. cerevisiae* (Gamalinda et al., 2013) especially once grown on glucose. Induced abundance of Rpl17 upon exposure to SMC may suggest that it modulates *A. fumigatus* response to harsh conditions depending on a carbon source. Several differentially expressed proteins were involved in carbohydrate metabolism and energy conversion, including isocitrate lyase AcuD (AFUA_4G13510) in proteome of ISSFT-021-30. AcuD is one of the key enzymes in glyoxylate cycle, which facilitates fungal growth on alternative C_2_ carbon sources (De Lucas et al., 1999). In addition, AcuE, another enzyme in the glyoxylate cycle, exhibited increased abundance in ISS-isolated ISSFT-021 and IF1SW-F4 when compared to clinical isolates Af293 and CEA10 (Blachowicz et al., 2019). Further, increased abundance of proteins involved in starvation response was observed in the ISS-isolated JSC-093350089 *A. niger* when compared to the well-studied culture collection strain ATCC 1015 (Romsdahl et al., 2018). These findings suggest that increased production of starvation-response enzymes play a role in the adaptation to space conditions. Several enzymes involved in chitin recognition and degradation were up-regulated in IMV 00236-30. These enzymes enable using chitinous debris as an alternative carbon source and allow morphogenetic changes during growth and differentiation (Gooday et al., 1986), which further suggests that alterations in carbohydrate metabolism are an adaptive response to SMC. Interestingly, when protein patterns of *Cryomyces antarcticus*, *Knufia perforans*, and *Exophiala jeanselmei* exposed in multilayers to SMC were analyzed by 2D gel electrophoresis no additional stress-induced proteins were observed (Zakharova et al., 2014).

This study affirms the enormous capability of filamentous fungi to adapt to extreme environmental conditions and thrive in a wide variety of ecological niches. To our knowledge, this is the first report of shotgun proteomic and metabolomic analyses of filamentous fungi in response to SMC. Such a complex state of the art analyses of fungal adaptive responses to space conditions are essential for ensuring safety in the era of future outer space explorations, as fungi will undoubtedly accompany people during space voyages. Thorough understanding of how filamentous fungi adapt to space conditions is important for both maintaining crew health and preventing biocorrosion of the spacecraft, as both opportunistic pathogenic fungi (Knox et al., 2016) and technophiles (Alekhova et al., 2005) have been reported on board of the ISS and Mir space stations.

## Materials and Methods

### Sample Collection Sites

Subcultures of the isolated strains were obtained from the Institute of Microbiology and Virology, Ukrainian Academy of Sciences, within the framework of a multiyear collaborative research program, to the Center for Environmental Biotechnology at Lawrence Berkeley National Laboratory (LBNL). For this study 12 Chernobyl nuclear accident-associated isolates were selected (Table 1).

### Preparation of Aluminum Coupons With Monolayers of Dried Fungal Conidia

High-grade aluminum coupons (Al 6061-T6) were precision cleaned for sterility as previously described (Osman et al., 2008). Each coupon was seeded with 100 μL of conidia suspension to contain ∼10^5^ conidia per coupon. Conidia were counted using a hemocytometer (Double Neubauer Counting Chamber, Hausser Scientific, Horsham, PA, United States) after harvesting 5 days grown cultures at 26°C on potato dextrose agar (PDA). Conidial suspensions were diluted in molecular biology grade water (Fisher Scientific, Waltham, MA, United States) and ∼10^5^ conidia were added to each coupon followed by drying overnight at the room temperature in a bio-hood. The monolayers of conidia were confirmed by scanning electron microscopy (data not shown).

### UV-C Exposure and Recovery

Aluminum coupons with dried fungal conidia were placed in a plastic Petri dish, without a lid, and exposed to UV-C using a low-pressure handheld mercury arc UV lamp (model UVG-11; UVP Inc., Upland, CA, United States). The lamp was placed above the sample, and the UV flux at the surface of exposed sample was measured using UVX digital radiometer (UVP Inc.). The exposure time required to produce doses: 0, 50, 100, 500, 1000, and 2,000 J/m^2^ was calculated at 100 μW cm^-2^. After exposure to UV-C 100 μL of 10% polyvinyl alcohol (PVA) was applied on each coupon and dried at 37°C for 50 min. Dried PVA along with fungal conidia was peeled using sterile forceps and added to 1 mL of molecular biology grade water (Fisher Scientific). The PVA extraction step was repeated. When PVA was dissolved, serial dilutions were prepared and plated on PDA in duplicates. Colony forming units (CFUs) were counted after 7 days of incubation at 26°C.

### Simulated Martian Conditions (SMC)

**FIGURE 6:**
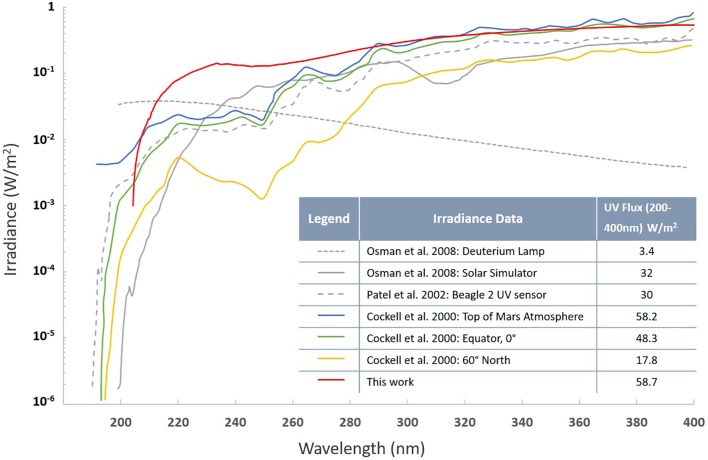
UV spectra (200–400 nm) of the solar simulator employed in this study. Other lighting spectra of Mars models are presented along with the integrated irradiance over the wavelength range from 200 to 400 nm.

Survival and response of fungal strains under SMC were tested in a Mars simulation chamber equipped with a UV transparent fused silica window according to a previously described set up (Garry et al., 2006; Osman et al., 2008; Peeters et al., 2010). Coupons prepared following the protocol described above were placed in the sterile Falcon tubes and sent to Netherlands. After arrival (∼1.5 weeks) samples deposited on aluminum coupons were placed inside the simulation chamber and subsequently the chamber was evacuated *via* an oil-free scroll pump (XDS5, Edwards Vacuum, Crawley, United Kingdom) to reach a base pressure of 20 Pa. While continuously being pumped, the chamber was purged five times with high purity CO_2_ (99.995%, H_2_0 < 5 ppm, O_2_ < 5 ppm, Praxair, Danbury, CT, United States), before establishing a continuous CO_2_ gas flow to maintain a stable chamber pressure of approximately 600 Pa. Samples were exposed at room temperature to simulated solar light (SF150 with a xenon arc lamp, 150W, Sciencetech Inc., London, Canada) *via* the fused silica window of the simulation chamber. The 200–400 nm wavelength integrated irradiance at the sample distance was 58.7 W/m^2^ (Figure 6). Samples were exposed for 5 and 30 min to cumulative doses of 2,670 and 16,110 J/m^2^, respectively, before being removed from the chamber and placed in sterile Falcon tubes for shipment back to JPL (Pasadena, CA, United States) for further analysis. Upon return to JPL, SMC-exposed samples were processed following the aforementioned PVA protocol to assess the survival rates.

### Secondary Metabolite Extraction and Analysis

*Aspergillus fumigatus* strains were cultivated at 30°C on GMM agar plates while *C. cladosporioides* strains were grown at 26°C on MEA agar plates starting with 10^7^ conidia/per Petri plate (*D* = 10 cm). After 5 days, agar was chopped, extracted with 25 mL methanol, and 25 mL of 1:1 methanol/dichloromethane each followed by 1 h sonication and filtration. After the second filtration, combined crude extracts from each isolate were evaporated in *vacuo*, suspended in 20 mL of water and partitioned with ethylacetate (20 mL). The ethylacetate layer was evaporated in *vacuo*, re-dissolved in 1 mL of 20% dimethyl sulfoxide/methanol and 10 μL was examined by high performance liquid chromatography-photodiode array detection-mass spectrometry (HPLC-DAD-MS) analysis. HPLC-MS was carried out using ThermoFinnigan LCQ Advantage ion trap mass spectrometer with an RP C_18_ column (Alltech Prevail C_18_ 3 mm 2.1 × 100 mm) at a flow rate 125 μL/min. The solvent gradient for LC/MS was 95% acetonitrile/H_2_O (solvent B) in 5% acetonitrile/H_2_O (solvent A) both containing 0.05% formic acid, as follows: 0% solvent B from 0 to 5 min, 0 to 100% solvent B from 5 to 35 min, 100% solvent B from 35 to 40 min, 100 to 0% solvent B from 40 to 45 min, and re-equilibration with 0% solvent B from 45 to 50 min.

### Proteome Samples Extraction and Processing

Exposure of fungal conidia to SMC required using ∼10^6^ conidia/coupon to avoid the shadowing effect (Osman et al., 2008), however such amount of biomass was not enough to perform detailed proteome analyses. Therefore, to observe permanent alterations in proteomes of SMC-exposed strains when compared to unexposed ones *A. fumigatus* strains were regrown at 30°C on GMM and *C. cladosporioides* strains at 26°C on MEA starting with 10^7^ conidia/per Petri plate (*D* = 10 cm). After 5 days, mycelia and spores from agar plates were collected and stored at -80 °C prior to protein extraction at City of Hope (Duarte, CA, United States). The protein was extracted as previously described (Romsdahl et al., 2018). In brief, mycelia, and spores were lysed and homogenized using a bead beater. Protein concentrations were measured by Bradford assay with a bovine serum albumin standard curve (Bio-Rad Laboratories, Inc., Hercules, CA, United States).

The samples were processed for a tandem mass tag (TMT) labeling as described by Romsdahl et al. (2018) with modification. The proteomic profiling of *A. fumigatus* and *C. cladosporioides* strains was carried out in two separate TMT LC/MS experiments. *A. fumigatus* strains ISSFT-021 and ISSFT-021-30 were labeled with TMT^6^-128 and TMT^6^-129, respectively. Two biological samples of *C. cladosporioides* strains IMV00 236 and IMV00 236-30 were labeled with TMT^6^-128/130 and TMT^6^-129/131, respectively.

The samples were analyzed on an Orbitrap Fusion Tribrid mass spectrometer with an EASY-nLC 1000 Liquid Chromatograph, a 75 μm × 2 cm Acclaim PepMap100 C_18_ trapping column, and a 75 μm × 25 cm PepMap RSLC C_18_ analytical column, and an Easy-Spray ion source (Thermo Fisher Scientific) as previously described (Romsdahl et al., 2018).

### Quantitative Proteomics Analysis

All MS spectra were analyzed using Proteome Discoverer (version 2.2.0.388, Thermo Fisher Scientific) with Sequest-HT search engines. Protein databases were either *A. fumigatus* Af293 database from NCBI containing 9845 non-redundant sequences or an in-house annotated draft genome sequence of *C. cladosporioides* (MSJH00000000). The search parameters were described by Romsdahl et al. (2018).

Technical triplicate measurements for each protein were averaged. Only proteins that were identified with at least one peptide detected in each technical replicate, and quantified in all technical and biological replicates, were considered for the analysis. The identified proteins were then averaged and Log2 transformed. Student *t*-test was performed to identify proteins that are differentially expressed between each SMC-exposed and unexposed group. Proteins with *p*-value ≤ 0.05 were further evaluated for up- and down-regulation using a cut-off value of ≥±2-fold change. AspGD gene ontology (GO) Slim terms (Cerqueira et al., 2014) were used to study the distribution of differentially expressed proteins among biological processes in SMC-exposed ISSFT-021-30 while the cluster of orthologous genes (COG) database (Tatusov et al., 2000) used in CloVR (Angiuoli et al., 2011) was used to study the distribution of differentially expressed proteins in SMC-exposed IMV 00236-30.

### Genome Annotation

Genome annotation of *C. cladosporioides* IMV 00236 was performed on the deposited assembly (MSJH00000000) with Funannotate (v1.5.1) (Love et al., 2018). Proteins from *Capnodiales* fungi (*Dothideomycetes*), Swissprot database (Swiss Institute of Bioinformatics Members [SIB], 2016), and transcripts from *Cladosporium sphaerospermum* UM 843 (Ng et al., 2012) were used as informant sequences. Conserved genes were identified from BUSCO core set “ascomycota_odb9” and were used to create training set for *ab initio* gene prediction by Augustus (Stanke et al., 2006; Simão et al., 2015). The *ab initio* predictor GenemarkHMM-ES was trained using its self-training procedures (Ter-Hovhannisyan et al., 2008). These predictions along with splice-aware aligned proteins using DIAMOND (Buchfink et al., 2015) followed by refinement with exonerate to improve spliced alignment accuracy (Slater and Birney, 2005). Consensus gene models were generated from the combined evidence with Evidence Modeler (Haas et al., 2008). Predicted gene function from conserved protein domains (El-Gebali et al., 2018), Swissprot (Swiss Institute of Bioinformatics Members [SIB], 2016) and inferred homology to conserved protein clusters in eggNOGdb (Huerta-Cepas et al., 2018), and secondary metabolite cluster prediction (Blin et al., 2017).

## Author Contributions

AB drafted the manuscript, carried out the UV-C exposure experiments, and contributed to data analysis and interpretation and strain identification. AC and MK conducted protein sample processing, LC/MS analyses, and proteome data processing. AE and PE enabled the simulated Martian chamber at the Leiden Institute of Chemistry, conducted the SMC experiments, and drafted parts of the manuscript. JS annotated the genome of IMV 00236 for proteome analysis. TT provided Chernobyl isolated strains and conducted morphological characterization and identification. CW drafted the manuscript and interpreted the metabolomics data. KV designed the study, interpreted the data, and drafted and critically reviewed the manuscript. All authors read and approved the final manuscript.

## Conflict of Interest Statement

The authors declare that the research was conducted in the absence of any commercial or financial relationships that could be construed as a potential conflict of interest.
