# Supplementary material for: Proteomic and Metabolomic Characteristics of Extremophilic Fungi Under Simulated Mars Conditions
Source: Front Microbiol. 2019 May 15;10:1013. doi: 10.3389/fmicb.2019.01013 (PMC6529585; doi:10.3389/fmicb.2019.01013)
Supplement: Supplementary file 1 [file Data_Sheet_1.PDF]

Supplementary Table 1. Quantitative analysis of the UV-C tolerance of extremotolerant fungi isolated from Chernobyl explosion sites and the International Space Station

	Growth after exposure of fungal conidia to UV-C (254 nm)[b]					
Strain[a]	No UV-C	100 J/m^2	500 J/m^2	1000 J/m^2	2000 J/m^2	Percent of survival 2000 J/m^2 dose
IMV 00034*	+	+	+	+	+	0.02
IMV 00045*	+	+	+	+	+	3.48
IMV 00236*	+	+	+	+	+	3.60
IMV 00253*	+	+	+	+	+	0.17
IMV 00265	+	+	+	+	-	0.00
IMV 00293	+	+	+	+	-	0.00
IMV 00454	+	+	+	+	+	0.50
IMV 00738	+	+	+	+	+	2.18
IMV 00882	+	+	+	+	-	0.00
IMV 01167	+	+	+	+	+	0.02
IMV 01221	+	+	+	+	+	0.12
IMV 01851	+	+	+	+	+	0.08
ISSFT-021	+	+	+	+	+	0.19

a – **IMV** - Institute for Microbiology and Virology (Academy of Sciences), Kiyv, Ukraine,
ISSFT - International Space Station Filter,
***** radiotropism.

b – "+" growth after exposure,
"-" no growth after exposure.

Supplementary Table ST2. Differentially abundant proteins in SMC-exposed ISSFT-021-30

Accession	ORF	Function / activity	ISSFT-021-30/ISSFT-021 log2FC	p-value
70998694	AFUA_5G05630	60S ribosomal protein L23	2.155	1.19E-03
70984072	AFUA_6G05200	60S ribosomal protein L28	2.012	4.67E-02
70993266	AFUA_4G13510	isocitrate lyase AcuD	1.781	4.60E-03
70994280	AFUA_4G08480	26S proteasome regulatory subunit Rpn2	1.693	5.19E-05
70982093	AFUA_4G03880	60S ribosomal protein L7	1.658	7.86E-03
70994610	AFUA_4G07435	60S ribosomal protein L36	1.598	2.68E-04
70998550	AFUA_5G06360	60S ribosomal protein L8	1.589	1.46E-03
70989247	AFUA_2G03380	alkaline serine protease	1.581	2.37E-03
70994548	AFUA_4G07730	60S ribosomal protein L11	1.559	3.32E-02
146322400	AFUA_1G03390	60S ribosomal protein L12	1.536	4.06E-03
70993286	AFUA_4G13410	37S ribosomal protein Rsm24	1.527	2.17E-02
71000719	AFUA_3G05600	60S ribosomal protein L27a	1.485	2.33E-02
70995022	AFUA_1G09100	60S ribosomal protein L9	1.469	1.38E-03
70992323	AFUA_6G11260	ribosomal protein L26	1.462	6.21E-03
70995824	AFUA_1G12970	conserved hypothetical protein	1.455	4.85E-02
70985070	AFUA_5G03020	60S ribosomal protein L4	1.426	3.84E-02
70992391	AFUA_6G11610	1,4-beta-D-glucan-cellobiohydrolyase	1.416	3.85E-03
70997938	AFUA_5G09380	conserved hypothetical protein	1.414	7.66E-04
70998408	AFUA_5G07080	beta-glucosidase	1.392	4.37E-02
71001672	AFUA_2G11850	60S ribosomal protein L3	1.389	2.36E-04
71002584	AFUA_2G16370	60S ribosomal protein L32	1.389	4.93E-03
70993340	AFUA_4G13140	conserved hypothetical protein	1.374	9.96E-03
70996112	AFUA_1G14410	60S ribosomal protein L17	1.363	5.82E-03
71001148	AFUA_2G09210	60S ribosomal protein L10	1.339	1.17E-02
70989179	AFUA_2G03040	ribosomal protein L34 protein	1.338	4.84E-02
70983059	AFUA_8G01490	endoglucanase	1.318	1.64E-02
71000493	AFUA_3G06760	60S ribosomal protein L37	1.317	1.48E-02
70990816	AFUA_1G05080	60S ribosomal protein P0	1.265	5.66E-03
146323659	AFUA_4G04460	60S ribosomal protein L13	1.253	5.15E-03
70999105	AFUA_3G13480	translation initiation factor 2 alpha subunit	1.224	3.30E-02
70984360	AFUA_6G03830	ribosomal protein L14	1.219	3.22E-03
70984653	AFUA_6G02440	60S ribosomal protein L24a	1.218	1.33E-03
70987156	AFUA_7G04520	conserved hypothetical protein	1.215	5.74E-03
70992591	AFUA_6G12660	40S ribosomal protein S10b	1.184	2.46E-02
70995446	AFUA_1G11130	60S ribosomal protein L6	1.184	2.20E-02
146322664	AFUA_1G16523	40S ribosomal protein S25	1.170	3.39E-03
70995808	AFUA_1G12890	60S ribosomal protein L5	1.165	1.89E-03
70996048	AFUA_1G14090	histidinol-phosphate aminotransferase	1.145	5.56E-03
71000010	AFUA_3G08940	proteasome regulatory particle subunit (RpnL)	1.141	2.59E-03
71000786	AFUA_2G07380	60S ribosomal protein L18	1.103	5.32E-03
71001146	AFUA_2G09200	60S ribosomal protein L30	1.099	1.83E-03
71002676	AFUA_2G16820	curved DNA-binding protein (42 kDa protein)	1.097	1.32E-03
146323370	AFUA_3G08930	tripeptidyl peptidase SED3	1.091	4.30E-02
70993620	AFUA_4G11730	glycerol dehydrogenase (GldB)	1.082	3.70E-02
71002510	AFUA_2G16010	prolyl-tRNA synthetase	1.071	2.97E-02
70989285	AFUA_2G03590	40S ribosomal protein S21	1.053	8.30E-04
70995576	AFUA_1G11770	COPII-coated vesicle protein SurF4/Erv29	1.033	2.50E-02
70995281	AFUA_1G10350	phosphoglycerate kinase PgkA	1.027	4.74E-03
70981726	AFUA_4G00640	macrophomate synthase	1.018	3.22E-02
70989741	AFUA_2G05910	hexokinase Kxk	1.010	1.07E-02
70991192	AFUA_1G06960	pyruvate dehydrogenase E1 component alpha subunit	1.004	3.76E-02
146323064	AFUA_2G16400	translation initiation factor 4B	-1.048	4.36E-02
70993154	AFUA_4G14070	glycosyl transferase	-1.080	2.65E-02
71001966	AFUA_2G13310	RING finger domain protein	-1.178	1.93E-02
70984070	AFUA_6G05210	malate dehydrogenase, NAD-dependent	-1.182	1.36E-04
70991893	AFUA_6G09070	3'(2'),5'-bisphosphate nucleotidase	-1.206	3.65E-02

70983536	AFUA_8G04890	conserved hypothetical protein	-1.209	4.06E-02
70998160	AFUA_5G08270	HAD superfamily hydrolase	-1.211	4.06E-04
70998574	AFUA_5G06240	alcohol dehydrogenase	-1.225	1.83E-04
70996316	AFUA_1G15450	adenylosuccinate synthetase AdB	-1.309	4.62E-03
70985144	AFUA_5G02640	O-methyltransferase	-1.317	4.04E-04
70998156	AFUA_5G08290	aldo-keto reductase	-1.323	1.04E-02
146324524	AFUA_6G13330	RNA binding protein	-1.360	2.27E-03
70982594	AFUA_7G01060	cysteine-rich secreted protein	-1.376	2.56E-02
70998364	AFUA_5G07300	electron transfer flavoprotein, beta subunit	-1.389	1.62E-02
146323042	AFUA_2G15510	DUF866 domain protein	-1.419	3.56E-02
70994846	AFUA_4G06240	progesterone binding protein	-1.456	3.97E-02
71002834	AFUA_2G17630	conserved hypothetical protein	-1.550	3.56E-02
70990780	AFUA_1G04900	proliferating cell nuclear antigen (PCNA)	-1.567	4.26E-03
70987183	AFUA_7G04380	alcohol dehydrogenase	-1.601	1.13E-04
70989906	AFUA_1G00500	FMN dependent dehydrogenase	-1.938	2.91E-02
70994328	AFUA_4G08410	mannose-6-phosphate isomerase, class I	-2.230	1.53E-02
70994706	AFUA_4G06960	UBX domain protein	-2.271	8.23E-04
70999410	AFUA_3G11970	C2H2 transcription factor PacC	-2.374	3.62E-02
70988771	AFUA_2G01040	formaldehyde dehydrogenase	-2.674	8.06E-04

* Log2 fold change of ISSFT-021-30 min compared to unexposed ISSFT-021 counterpart ($P < 0.05$)

Supplementary Table ST3a. Significantly over-represented up-regulated biological processes in SMC-exposed ISSFT-021-30

GO ID	GO Term	Genes in the bkgd with this term	Genes in your result with this term	Percent of bkgd Genes in your result	Fold enrichment	Odds ratio	P-value	Benjamini	Bonferroni
GO:0006412	translation	202	26	12.9	24.73	56.56	4.55E-31	1.95E-28	1.95E-28
GO:0043043	peptide biosynthetic process	252	26	10.3	19.82	43.81	1.78E-28	3.82E-26	7.64E-26
GO:0006518	peptide metabolic process	270	26	9.6	18.5	40.5	1.12E-27	1.61E-25	4.82E-25
GO:0043604	amide biosynthetic process	299	26	8.7	16.71	36.09	1.68E-26	1.80E-24	7.19E-24
GO:0043603	cellular amide metabolic process	337	26	7.7	14.82	31.55	3.89E-25	3.34E-23	1.67E-22
GO:1901566	organonitrogen compound biosynthetic process	662	30	4.5	8.71	20.6	1.23E-22	8.80E-21	5.28E-20
GO:0044271	cellular nitrogen compound biosynthetic process	818	29	3.5	6.81	14.97	8.78E-19	5.38E-17	3.77E-16
GO:0044267	cellular protein metabolic process	769	27	3.5	6.75	13.65	3.49E-17	1.87E-15	1.50E-14
GO:0019538	protein metabolic process	858	28	3.3	6.27	13.08	4.45E-17	2.12E-15	1.91E-14
GO:0034645	cellular macromolecule biosynthetic process	708	26	3.7	7.06	13.82	6.07E-17	2.60E-15	2.60E-14
GO:0009059	macromolecule biosynthetic process	714	26	3.6	7	13.69	7.47E-17	2.91E-15	3.20E-14
GO:0010467	gene expression	800	26	3.3	6.24	12.06	1.20E-15	4.30E-14	5.16E-13
GO:1901564	organonitrogen compound metabolic process	1435	32	2.2	4.28	10.02	4.19E-15	1.38E-13	1.80E-12
GO:0044249	cellular biosynthetic process	1309	30	2.3	4.4	9.46	2.96E-14	9.08E-13	1.27E-11
GO:1901576	organic substance biosynthetic process	1330	30	2.3	4.33	9.28	4.57E-14	1.31E-12	1.96E-11
GO:0000027	ribosomal large subunit assembly	22	8	36.4	69.86	129.34	9.18E-14	2.46E-12	3.94E-11
GO:0009058	biosynthetic process	1378	30	2.2	4.18	8.9	1.19E-13	3.01E-12	5.12E-11
GO:0034641	cellular nitrogen compound metabolic process	1446	30	2.1	3.99	8.4	4.36E-13	1.04E-11	1.87E-10
GO:0044238	primary metabolic process	2587	37	1.4	2.75	7.46	7.04E-12	1.59E-10	3.02E-09
GO:0005975	carbohydrate metabolic process	438	8	1.8	3.51	4.03	1.75E-03	1.19E-02	7.52E-01
GO:0042255	ribosome assembly	37	8	21.6	41.54	62.34	1.04E-11	2.24E-10	4.48E-09
GO:0044260	cellular macromolecule metabolic process	1395	28	2	3.86	7.46	1.10E-11	2.24E-10	4.71E-09
GO:0071826	ribonucleoprotein complex subunit organization	65	9	13.8	26.6	37.08	3.43E-11	6.68E-10	1.47E-08
GO:0043170	macromolecule metabolic process	1759	30	1.7	3.28	6.62	7.63E-11	1.42E-09	3.27E-08
GO:0042273	ribosomal large subunit biogenesis	50	8	16	30.74	42.99	1.38E-10	2.47E-09	5.92E-08
GO:0071704	organic substance metabolic process	2912	37	1.3	2.44	6.32	3.11E-10	5.33E-09	1.33E-07
GO:0044237	cellular metabolic process	2648	35	1.3	2.54	5.97	6.40E-10	1.06E-08	2.74E-07
GO:0042254	ribosome biogenesis	168	11	6.5	12.58	16.8	7.14E-10	1.13E-08	3.06E-07
GO:0006807	nitrogen compound metabolic process	2208	32	1.4	2.78	5.86	7.87E-10	1.21E-08	3.38E-07
GO:0022618	ribonucleoprotein complex assembly	62	8	12.9	24.79	33.4	8.30E-10	1.23E-08	3.56E-07
GO:0002181	cytoplasmic translation	22	6	27.3	52.4	81.09	1.03E-09	1.47E-08	4.41E-07
GO:0022613	ribonucleoprotein complex biogenesis	192	11	5.7	11.01	14.53	2.95E-09	4.09E-08	1.27E-06
GO:0070925	organelle assembly	77	8	10.4	19.96	26.1	4.87E-09	6.53E-08	2.09E-06
GO:0009987	cellular process	3525	38	1.1	2.07	5.25	2.19E-08	2.85E-07	9.39E-06
GO:0008152	metabolic process	3751	38	1	1.95	4.75	1.49E-07	1.88E-06	6.40E-05
GO:0043933	protein-containing complex subunit organization	276	9	3.3	6.26	7.61	1.06E-05	1.29E-04	4.53E-03
GO:0034622	cellular protein-containing complex assembly	223	8	3.6	6.89	8.25	1.74E-05	2.07E-04	7.47E-03
GO:0006096	glycolytic process	11	3	27.3	52.4	76.09	2.13E-05	2.34E-04	9.13E-03
GO:0042866	pyruvate biosynthetic process	11	3	27.3	52.4	76.09	2.13E-05	2.34E-04	9.13E-03
GO:0006757	ATP generation from ADP	11	3	27.3	52.4	76.09	2.13E-05	2.34E-04	9.13E-03
GO:0065003	protein-containing complex assembly	234	8	3.4	6.57	7.84	2.46E-05	2.64E-04	1.06E-02
GO:0006165	nucleoside diphosphate phosphorylation	12	3	25	48.03	67.62	2.83E-05	2.96E-04	1.21E-02
GO:0044085	cellular component biogenesis	516	11	2.1	4.1	5.03	5.41E-05	5.53E-04	2.32E-02
GO:0046939	nucleotide phosphorylation	15	3	20	38.42	50.7	5.78E-05	5.77E-04	2.48E-02
GO:0046031	ADP metabolic process	16	3	18.8	36.02	46.8	7.09E-05	6.92E-04	3.04E-02
GO:0009135	purine nucleoside diphosphate metabolic process	17	3	17.6	33.9	43.45	8.58E-05	7.83E-04	3.68E-02
GO:0009185	ribonucleoside diphosphate metabolic process	17	3	17.6	33.9	43.45	8.58E-05	7.83E-04	3.68E-02
GO:0009179	purine ribonucleoside diphosphate metabolic process	17	3	17.6	33.9	43.45	8.58E-05	7.83E-04	3.68E-02
GO:0009132	nucleoside diphosphate metabolic process	20	3	15	28.82	35.77	1.42E-04	1.27E-03	6.10E-02
GO:0006090	pyruvate metabolic process	21	3	14.3	27.45	33.78	1.65E-04	1.45E-03	7.10E-02
GO:0009166	nucleotide catabolic process	23	3	13	25.06	30.4	2.19E-04	1.88E-03	9.38E-02
GO:1901292	nucleoside phosphate catabolic process	24	3	12.5	24.01	28.95	2.49E-04	2.09E-03	1.07E-01
GO:0019359	nicotinamide nucleotide biosynthetic process	26	3	11.5	22.17	26.42	3.17E-04	2.57E-03	1.36E-01
GO:0019363	pyridine nucleotide biosynthetic process	26	3	11.5	22.17	26.42	3.17E-04	2.57E-03	1.36E-01

GO:0022607	cellular component assembly	353	8	2.3	4.35	5.07	4.30E-04	3.42E-03	1.85E-01
GO:0006754	ATP biosynthetic process	29	3	10.3	19.87	23.37	4.41E-04	3.44E-03	1.89E-01
GO:0009206	purine ribonucleoside triphosphate biosynthetic process	30	3	10	19.21	22.5	4.89E-04	3.68E-03	2.10E-01
GO:0009145	purine nucleoside triphosphate biosynthetic process	30	3	10	19.21	22.5	4.89E-04	3.68E-03	2.10E-01
GO:0009201	ribonucleoside triphosphate biosynthetic process	31	3	9.7	18.59	21.69	5.39E-04	3.99E-03	2.31E-01
GO:0009142	nucleoside triphosphate biosynthetic process	32	3	9.4	18.01	20.94	5.92E-04	4.24E-03	2.54E-01
GO:0072525	pyridine-containing compound biosynthetic process	32	3	9.4	18.01	20.94	5.92E-04	4.24E-03	2.54E-01
GO:0034404	nucleobase-containing small molecule biosynthetic process	35	3	8.6	16.47	18.97	7.73E-04	5.44E-03	3.32E-01
GO:0046434	organophosphate catabolic process	36	3	8.3	16.01	18.4	8.40E-04	5.82E-03	3.61E-01
GO:0009168	purine ribonucleoside monophosphate biosynthetic process	48	3	6.3	12.01	13.47	1.95E-03	1.29E-02	8.36E-01
GO:0009127	purine nucleoside monophosphate biosynthetic process	48	3	6.3	12.01	13.47	1.95E-03	1.29E-02	8.36E-01
GO:0009152	purine ribonucleotide biosynthetic process	49	3	6.1	11.76	13.18	2.07E-03	1.34E-02	8.87E-01
GO:0006164	purine nucleotide biosynthetic process	50	3	6	11.53	12.9	2.19E-03	1.38E-02	9.40E-01
GO:0071840	cellular component organization or biogenesis	1048	13	1.2	2.38	2.88	2.19E-03	1.38E-02	9.42E-01
GO:0009156	ribonucleoside monophosphate biosynthetic process	51	3	5.9	11.3	12.63	2.32E-03	1.44E-02	9.96E-01
GO:0009260	ribonucleotide biosynthetic process	53	3	5.7	10.87	12.12	2.59E-03	1.54E-02	1.00E+00
GO:0009124	nucleoside monophosphate biosynthetic process	53	3	5.7	10.87	12.12	2.59E-03	1.54E-02	1.00E+00
GO:0046034	ATP metabolic process	53	3	5.7	10.87	12.12	2.59E-03	1.54E-02	1.00E+00
GO:0019362	pyridine nucleotide metabolic process	54	3	5.6	10.67	11.88	2.73E-03	1.58E-02	1.00E+00
GO:0046496	nicotinamide nucleotide metabolic process	54	3	5.6	10.67	11.88	2.73E-03	1.58E-02	1.00E+00
GO:0072522	purine-containing compound biosynthetic process	57	3	5.3	10.11	11.22	3.19E-03	1.80E-02	1.00E+00
GO:0046390	ribose phosphate biosynthetic process	57	3	5.3	10.11	11.22	3.19E-03	1.80E-02	1.00E+00
GO:0009205	purine ribonucleoside triphosphate metabolic process	59	3	5.1	9.77	10.82	3.52E-03	1.96E-02	1.00E+00
GO:0009144	purine nucleoside triphosphate metabolic process	60	3	5	9.61	10.62	3.69E-03	2.00E-02	1.00E+00
GO:0009199	ribonucleoside triphosphate metabolic process	60	3	5	9.61	10.62	3.69E-03	2.00E-02	1.00E+00
GO:0072524	pyridine-containing compound metabolic process	61	3	4.9	9.45	10.44	3.87E-03	2.07E-02	1.00E+00
GO:0015976	carbon utilization	19	2	10.5	20.22	23.36	4.29E-03	2.27E-02	1.00E+00
GO:0006733	oxidoreduction coenzyme metabolic process	65	3	4.6	8.87	9.76	4.62E-03	2.39E-02	1.00E+00
GO:0009141	nucleoside triphosphate metabolic process	65	3	4.6	8.87	9.76	4.62E-03	2.39E-02	1.00E+00
GO:0050686	negative regulation of mRNA processing	1	1	100	192.12	inf	5.21E-03	2.59E-02	1.00E+00
GO:0048025	negative regulation of mRNA splicing, via spliceosome	1	1	100	192.12	inf	5.21E-03	2.59E-02	1.00E+00
GO:0033119	negative regulation of RNA splicing	1	1	100	192.12	inf	5.21E-03	2.59E-02	1.00E+00
GO:0034655	nucleobase-containing compound catabolic process	68	3	4.4	8.48	9.31	5.25E-03	2.59E-02	1.00E+00
GO:0009126	purine nucleoside monophosphate metabolic process	74	3	4.1	7.79	8.52	6.64E-03	3.20E-02	1.00E+00
GO:0009167	purine ribonucleoside monophosphate metabolic process	74	3	4.1	7.79	8.52	6.64E-03	3.20E-02	1.00E+00
GO:0032984	protein-containing complex disassembly	24	2	8.3	16.01	18.04	6.81E-03	3.25E-02	1.00E+00
GO:0016052	carbohydrate catabolic process	148	4	2.7	5.19	5.68	7.21E-03	3.40E-02	1.00E+00
GO:0009161	ribonucleoside monophosphate metabolic process	77	3	3.9	7.49	8.17	7.41E-03	3.46E-02	1.00E+00
GO:0009123	nucleoside monophosphate metabolic process	79	3	3.8	7.3	7.95	7.96E-03	3.67E-02	1.00E+00
GO:0009165	nucleotide biosynthetic process	80	3	3.8	7.2	7.85	8.24E-03	3.76E-02	1.00E+00
GO:0044262	cellular carbohydrate metabolic process	155	4	2.6	4.96	5.41	8.46E-03	3.80E-02	1.00E+00
GO:0006996	organelle organization	690	9	1.3	2.51	2.85	8.50E-03	3.80E-02	1.00E+00
GO:1901293	nucleoside phosphate biosynthetic process	84	3	3.6	6.86	7.46	9.42E-03	4.16E-02	1.00E+00

GO:0009150	purine ribonucleotide metabolic process	85	3	3.5	6.78	7.37	9.72E-03	4.16E-02	1.00E+00
GO:0006163	purine nucleotide metabolic process	86	3	3.5	6.7	7.28	1.00E-02	4.16E-02	1.00E+00
GO:1903312	negative regulation of mRNA metabolic process	2	1	50	96.06	194.92	1.04E-02	4.16E-02	1.00E+00
GO:0006973	intracellular accumulation of glycerol	2	1	50	96.06	194.92	1.04E-02	4.16E-02	1.00E+00
GO:0032445	fructose import	2	1	50	96.06	194.92	1.04E-02	4.16E-02	1.00E+00
GO:0035617	stress granule disassembly	2	1	50	96.06	194.92	1.04E-02	4.16E-02	1.00E+00
GO:0010045	response to nickel cation	2	1	50	96.06	194.92	1.04E-02	4.16E-02	1.00E+00
GO:0042867	pyruvate catabolic process	2	1	50	96.06	194.92	1.04E-02	4.16E-02	1.00E+00
GO:0000461	endonucleolytic cleavage to generate mature 3'-end of SSU-rRNA from (SSU-rRNA, 5.8S rRNA, LSU-rRNA)	2	1	50	96.06	194.92	1.04E-02	4.16E-02	1.00E+00
GO:0006433	prolyl-tRNA aminoacylation	2	1	50	96.06	194.92	1.04E-02	4.16E-02	1.00E+00
GO:0009259	ribonucleotide metabolic process	89	3	3.4	6.48	7.02	1.10E-02	4.38E-02	1.00E+00
GO:0006091	generation of precursor metabolites and energy	91	3	3.3	6.33	6.86	1.17E-02	4.61E-02	1.00E+00
GO:0046700	heterocycle catabolic process	92	3	3.3	6.26	6.78	1.21E-02	4.70E-02	1.00E+00
GO:0044270	cellular nitrogen compound catabolic process	97	3	3.1	5.94	6.42	1.39E-02	5.37E-02	1.00E+00
GO:0043561	regulation of translational initiation in response to osmotic stress	3	1	33.3	64.04	97.45	1.55E-02	5.55E-02	1.00E+00
GO:0048024	regulation of mRNA splicing, via spliceosome	3	1	33.3	64.04	97.45	1.55E-02	5.55E-02	1.00E+00
GO:0015755	fructose transmembrane transport	3	1	33.3	64.04	97.45	1.55E-02	5.55E-02	1.00E+00
GO:0006002	fructose 6-phosphate metabolic process	3	1	33.3	64.04	97.45	1.55E-02	5.55E-02	1.00E+00
GO:0043484	regulation of RNA splicing	3	1	33.3	64.04	97.45	1.55E-02	5.55E-02	1.00E+00
GO:0050826	response to freezing	3	1	33.3	64.04	97.45	1.55E-02	5.55E-02	1.00E+00
GO:0050684	regulation of mRNA processing	3	1	33.3	64.04	97.45	1.55E-02	5.55E-02	1.00E+00
GO:0046835	carbohydrate phosphorylation	3	1	33.3	64.04	97.45	1.55E-02	5.55E-02	1.00E+00
GO:0071497	cellular response to freezing	3	1	33.3	64.04	97.45	1.55E-02	5.55E-02	1.00E+00
GO:0032787	monocarboxylic acid metabolic process	188	4	2.1	4.09	4.42	1.63E-02	5.69E-02	1.00E+00
GO:0019439	aromatic compound catabolic process	103	3	2.9	5.6	6.03	1.63E-02	5.69E-02	1.00E+00
GO:0009108	coenzyme biosynthetic process	103	3	2.9	5.6	6.03	1.63E-02	5.69E-02	1.00E+00
GO:0072521	purine-containing compound metabolic process	104	3	2.9	5.54	5.97	1.67E-02	5.74E-02	1.00E+00
GO:0072330	monocarboxylic acid biosynthetic process	104	3	2.9	5.54	5.97	1.67E-02	5.74E-02	1.00E+00
GO:0016043	cellular component organization	913	10	1.1	2.1	2.39	1.77E-02	6.02E-02	1.00E+00
GO:0019318	hexose metabolic process	40	2	5	9.61	10.43	1.83E-02	6.17E-02	1.00E+00
GO:0019693	ribose phosphate metabolic process	109	3	2.8	5.29	5.68	1.89E-02	6.35E-02	1.00E+00
GO:0022411	cellular component disassembly	41	2	4.9	9.37	10.16	1.91E-02	6.36E-02	1.00E+00
GO:0006097	glyoxylate cycle	4	1	25	48.03	64.96	2.07E-02	6.52E-02	1.00E+00
GO:0043558	regulation of translational initiation in response to stress	4	1	25	48.03	64.96	2.07E-02	6.52E-02	1.00E+00
GO:0046487	glyoxylate metabolic process	4	1	25	48.03	64.96	2.07E-02	6.52E-02	1.00E+00
GO:0032988	ribonucleoprotein complex disassembly	4	1	25	48.03	64.96	2.07E-02	6.52E-02	1.00E+00
GO:0006000	fructose metabolic process	4	1	25	48.03	64.96	2.07E-02	6.52E-02	1.00E+00
GO:0043557	regulation of translation in response to osmotic stress	4	1	25	48.03	64.96	2.07E-02	6.52E-02	1.00E+00
GO:0006086	acetyl-CoA biosynthetic process from pyruvate	4	1	25	48.03	64.96	2.07E-02	6.52E-02	1.00E+00
GO:1901361	organic cyclic compound catabolic process	114	3	2.6	5.06	5.43	2.13E-02	6.68E-02	1.00E+00
GO:1904659	glucose transmembrane transport	5	1	20	38.42	48.72	2.58E-02	7.78E-02	1.00E+00
GO:0070417	cellular response to cold	5	1	20	38.42	48.72	2.58E-02	7.78E-02	1.00E+00
GO:0006013	mannose metabolic process	5	1	20	38.42	48.72	2.58E-02	7.78E-02	1.00E+00
GO:0006085	acetyl-CoA biosynthetic process	5	1	20	38.42	48.72	2.58E-02	7.78E-02	1.00E+00
GO:0046323	glucose import	5	1	20	38.42	48.72	2.58E-02	7.78E-02	1.00E+00
GO:0072329	monocarboxylic acid catabolic process	49	2	4.1	7.84	8.42	2.67E-02	8.02E-02	1.00E+00
GO:0019752	carboxylic acid metabolic process	450	6	1.3	2.56	2.79	2.85E-02	8.46E-02	1.00E+00
GO:0044042	glucan metabolic process	51	2	3.9	7.53	8.08	2.88E-02	8.46E-02	1.00E+00
GO:0005996	monosaccharide metabolic process	51	2	3.9	7.53	8.08	2.88E-02	8.46E-02	1.00E+00
GO:0006094	gluconeogenesis	6	1	16.7	32.02	38.97	3.08E-02	9.00E-02	1.00E+00
GO:0051188	cofactor biosynthetic process	133	3	2.3	4.33	4.62	3.17E-02	9.20E-02	1.00E+00
GO:0043436	oxoacid metabolic process	463	6	1.3	2.49	2.71	3.21E-02	9.25E-02	1.00E+00
GO:0006082	organic acid metabolic process	467	6	1.3	2.47	2.69	3.33E-02	9.52E-02	1.00E+00
GO:0046394	carboxylic acid biosynthetic process	237	4	1.7	3.24	3.48	3.44E-02	9.77E-02	1.00E+00
GO:0016053	organic acid biosynthetic process	238	4	1.7	3.23	3.46	3.48E-02	9.83E-02	1.00E+00
GO:0006415	translational termination	7	1	14.3	27.45	32.47	3.59E-02	9.93E-02	1.00E+00
GO:0035384	thioester biosynthetic process	7	1	14.3	27.45	32.47	3.59E-02	9.93E-02	1.00E+00

GO:0071616	acyl-CoA biosynthetic process	7	1	14.3	27.45	32.47	3.59E-02	9.93E-02	1.00E+00
GO:0006732	coenzyme metabolic process	147	3	2	3.92	4.17	4.08E-02	1.08E-01	1.00E+00
GO:0006547	histidine metabolic process	8	1	12.5	24.01	27.83	4.09E-02	1.08E-01	1.00E+00
GO:0045733	acetate catabolic process	8	1	12.5	24.01	27.83	4.09E-02	1.08E-01	1.00E+00
GO:0046364	monosaccharide biosynthetic process	8	1	12.5	24.01	27.83	4.09E-02	1.08E-01	1.00E+00
GO:0019319	hexose biosynthetic process	8	1	12.5	24.01	27.83	4.09E-02	1.08E-01	1.00E+00
GO:0052803	imidazole-containing compound metabolic process	8	1	12.5	24.01	27.83	4.09E-02	1.08E-01	1.00E+00
GO:0000105	histidine biosynthetic process	8	1	12.5	24.01	27.83	4.09E-02	1.08E-01	1.00E+00
GO:0008645	hexose transmembrane transport	8	1	12.5	24.01	27.83	4.09E-02	1.08E-01	1.00E+00
GO:0010411	xyloglucan metabolic process	9	1	11.1	21.35	24.35	4.59E-02	1.17E-01	1.00E+00
GO:0031125	rRNA 3'-end processing	9	1	11.1	21.35	24.35	4.59E-02	1.17E-01	1.00E+00
GO:1903311	regulation of mRNA metabolic process	9	1	11.1	21.35	24.35	4.59E-02	1.17E-01	1.00E+00
GO:0015749	monosaccharide transmembrane transport	9	1	11.1	21.35	24.35	4.59E-02	1.17E-01	1.00E+00
GO:0046015	regulation of transcription by glucose	9	1	11.1	21.35	24.35	4.59E-02	1.17E-01	1.00E+00
GO:0043555	regulation of translation in response to stress	9	1	11.1	21.35	24.35	4.59E-02	1.17E-01	1.00E+00
GO:0009117	nucleotide metabolic process	156	3	1.9	3.69	3.92	4.73E-02	1.19E-01	1.00E+00

Supplementary Table ST3b. Significantly over-represented down-regulated biological processes in SMC-exposed ISSFT-021-30

GO ID	GO Term	Genes in the bkgd with this term	Genes in your result with this term	Percent of bkgd Genes in your result	Fold enrichment	Odds ratio	P-value	Benjamini	Bonferroni
GO:0008152	metabolic process	3751	14	0.4	1.52	2.26	3.66E-02	1.11E-01	1.00E+00
GO:1901360	organic cyclic compound metabolic process	1396	7	0.5	2.05	2.49	4.49E-02	1.23E-01	1.00E+00
GO:0046483	heterocycle metabolic process	1295	7	0.5	2.21	2.71	3.13E-02	9.96E-02	1.00E+00
GO:0044281	small molecule metabolic process	825	7	0.8	3.46	4.51	2.83E-03	5.24E-02	7.27E-01
GO:0055114	oxidation-reduction process	714	6	0.8	3.43	4.27	6.34E-03	5.71E-02	1.00E+00
GO:0006082	organic acid metabolic process	467	5	1.1	4.37	5.3	4.82E-03	5.24E-02	1.00E+00
GO:0043436	oxoacid metabolic process	463	5	1.1	4.41	5.35	4.65E-03	5.24E-02	1.00E+00
GO:0019752	carboxylic acid metabolic process	450	5	1.1	4.54	5.52	4.11E-03	5.24E-02	1.00E+00
GO:1901135	carbohydrate derivative metabolic process	289	3	1	4.24	4.74	3.25E-02	1.02E-01	1.00E+00
GO:0017144	drug metabolic process	286	4	1.4	5.71	6.73	4.76E-03	5.24E-02	1.00E+00
GO:0019637	organophosphate metabolic process	278	3	1.1	4.41	4.93	2.94E-02	9.83E-02	1.00E+00
GO:0009628	response to abiotic stimulus	221	3	1.4	5.54	6.26	1.62E-02	7.69E-02	1.00E+00
GO:0055086	nucleobase-containing small molecule metabolic process	208	3	1.4	5.89	6.67	1.37E-02	7.36E-02	1.00E+00
GO:1901615	organic hydroxy compound metabolic process	185	3	1.6	6.62	7.53	1.00E-02	6.77E-02	1.00E+00
GO:0044282	small molecule catabolic process	141	2	1.4	5.79	6.3	4.62E-02	1.24E-01	1.00E+00
GO:0006790	sulfur compound metabolic process	139	3	2.2	8.81	10.12	4.55E-03	5.24E-02	1.00E+00
GO:0009636	response to toxic substance	120	2	1.7	6.8	7.44	3.44E-02	1.05E-01	1.00E+00
GO:1901617	organic hydroxy compound biosynthetic process	112	2	1.8	7.29	7.99	3.03E-02	9.87E-02	1.00E+00
GO:0104004	cellular response to environmental stimulus	111	2	1.8	7.36	8.06	2.98E-02	9.83E-02	1.00E+00
GO:0071214	cellular response to abiotic stimulus	111	2	1.8	7.36	8.06	2.98E-02	9.83E-02	1.00E+00
GO:0006066	alcohol metabolic process	109	3	2.8	11.24	13.03	2.29E-03	5.24E-02	5.88E-01
GO:0019693	ribose phosphate metabolic process	109	2	1.8	7.49	8.21	2.89E-02	9.83E-02	1.00E+00
GO:0072521	purine-containing compound metabolic process	104	2	1.9	7.85	8.62	2.65E-02	9.31E-02	1.00E+00
GO:0016054	organic acid catabolic process	101	2	2	8.08	8.88	2.51E-02	8.94E-02	1.00E+00
GO:0046395	carboxylic acid catabolic process	101	2	2	8.08	8.88	2.51E-02	8.94E-02	1.00E+00
GO:0016999	antibiotic metabolic process	93	3	3.2	13.17	15.37	1.45E-03	5.24E-02	3.73E-01
GO:0098754	detoxification	92	2	2.2	8.88	9.78	2.10E-02	8.25E-02	1.00E+00
GO:0006091	generation of precursor metabolites and energy	91	2	2.2	8.97	9.89	2.06E-02	8.25E-02	1.00E+00
GO:0009259	ribonucleotide metabolic process	89	2	2.2	9.17	10.12	1.98E-02	8.07E-02	1.00E+00
GO:0006163	purine nucleotide metabolic process	86	2	2.3	9.49	10.49	1.85E-02	8.06E-02	1.00E+00
GO:0009150	purine ribonucleotide metabolic process	85	2	2.4	9.61	10.61	1.81E-02	8.06E-02	1.00E+00
GO:0009123	nucleoside monophosphate metabolic process	79	2	2.5	10.34	11.45	1.58E-02	7.66E-02	1.00E+00
GO:0009161	ribonucleoside monophosphate metabolic process	77	2	2.6	10.6	11.76	1.50E-02	7.44E-02	1.00E+00
GO:0009126	purine nucleoside monophosphate metabolic process	74	2	2.7	11.03	12.25	1.39E-02	7.36E-02	1.00E+00
GO:0009167	purine ribonucleoside monophosphate metabolic process	74	2	2.7	11.03	12.25	1.39E-02	7.36E-02	1.00E+00
GO:0046165	alcohol biosynthetic process	65	2	3.1	12.56	14.01	1.09E-02	6.96E-02	1.00E+00
GO:0009063	cellular amino acid catabolic process	52	2	3.8	15.7	17.68	7.08E-03	5.71E-02	1.00E+00
GO:0043648	dicarboxylic acid metabolic process	32	2	6.3	25.52	29.53	2.73E-03	5.24E-02	7.01E-01
GO:0017001	antibiotic catabolic process	20	2	10	40.82	49.27	1.06E-03	5.24E-02	2.73E-01
GO:0046112	nucleobase biosynthetic process	20	1	5	20.41	22.32	4.79E-02	1.26E-01	1.00E+00
GO:0009132	nucleoside diphosphate metabolic process	20	1	5	20.41	22.32	4.79E-02	1.26E-01	1.00E+00
GO:0034308	primary alcohol metabolic process	19	2	10.5	42.97	52.18	9.59E-04	5.24E-02	2.46E-01
GO:0034644	cellular response to UV	19	1	5.3	21.49	23.57	4.56E-02	1.23E-01	1.00E+00
GO:0046164	alcohol catabolic process	18	1	5.6	22.68	24.95	4.32E-02	1.19E-01	1.00E+00
GO:0006301	postreplication repair	18	1	5.6	22.68	24.95	4.32E-02	1.19E-01	1.00E+00
GO:0009185	ribonucleoside diphosphate metabolic process	17	1	5.9	24.01	26.52	4.09E-02	1.15E-01	1.00E+00
GO:0009179	purine ribonucleoside diphosphate metabolic process	17	1	5.9	24.01	26.52	4.09E-02	1.15E-01	1.00E+00
GO:0009135	purine nucleoside diphosphate metabolic process	17	1	5.9	24.01	26.52	4.09E-02	1.15E-01	1.00E+00
GO:0033244	regulation of penicillin metabolic process	16	1	6.3	25.52	28.29	3.85E-02	1.12E-01	1.00E+00
GO:0006006	glucose metabolic process	16	1	6.3	25.52	28.29	3.85E-02	1.12E-01	1.00E+00
GO:0046031	ADP metabolic process	16	1	6.3	25.52	28.29	3.85E-02	1.12E-01	1.00E+00
GO:0034309	primary alcohol biosynthetic process	14	1	7.1	29.16	32.65	3.38E-02	1.05E-01	1.00E+00
GO:0006566	threonine metabolic process	13	1	7.7	31.4	35.37	3.14E-02	9.96E-02	1.00E+00
GO:0009113	purine nucleobase biosynthetic process	12	1	8.3	34.02	38.59	2.90E-02	9.83E-02	1.00E+00
GO:0006067	ethanol metabolic process	10	2	20	81.65	110.98	2.56E-04	5.24E-02	6.57E-02
GO:0009225	nucleotide-sugar metabolic process	10	1	10	40.82	47.17	2.42E-02	8.90E-02	1.00E+00
GO:1900196	regulation of penicillin biosynthetic process	10	1	10	40.82	47.17	2.42E-02	8.90E-02	1.00E+00

GO:0009068	aspartate family amino acid catabolic process	9	1	11.1	45.36	53.08	2.18E-02	8.25E-02	1.00E+00
GO:0046854	phosphatidylinositol phosphorylation	9	1	11.1	45.36	53.08	2.18E-02	8.25E-02	1.00E+00
GO:0046834	lipid phosphorylation	9	1	11.1	45.36	53.08	2.18E-02	8.25E-02	1.00E+00
GO:0046083	adenine metabolic process	8	1	12.5	51.03	60.66	1.94E-02	8.06E-02	1.00E+00
GO:0010446	response to alkaline pH	8	1	12.5	51.03	60.66	1.94E-02	8.06E-02	1.00E+00
GO:0071469	cellular response to alkaline pH	8	1	12.5	51.03	60.66	1.94E-02	8.06E-02	1.00E+00
GO:0006567	threonine catabolic process	8	1	12.5	51.03	60.66	1.94E-02	8.06E-02	1.00E+00
GO:0009226	nucleotide-sugar biosynthetic process	7	1	14.3	58.32	70.78	1.70E-02	7.81E-02	1.00E+00
GO:1900549	N',N'',N'''-triacetylfusarinine C metabolic process	7	1	14.3	58.32	70.78	1.70E-02	7.81E-02	1.00E+00
GO:0046084	adenine biosynthetic process	6	1	16.7	68.04	84.95	1.46E-02	7.36E-02	1.00E+00
GO:0046937	phytochelatin metabolic process	6	1	16.7	68.04	84.95	1.46E-02	7.36E-02	1.00E+00
GO:0046938	phytochelatin biosynthetic process	6	1	16.7	68.04	84.95	1.46E-02	7.36E-02	1.00E+00
GO:0006113	fermentation	5	1	20	81.65	106.2	1.22E-02	6.96E-02	1.00E+00
GO:0006106	fumarate metabolic process	5	1	20	81.65	106.2	1.22E-02	6.96E-02	1.00E+00
GO:0046185	aldehyde catabolic process	5	1	20	81.65	106.2	1.22E-02	6.96E-02	1.00E+00
GO:0070814	hydrogen sulfide biosynthetic process	5	1	20	81.65	106.2	1.22E-02	6.96E-02	1.00E+00
GO:0006115	ethanol biosynthetic process	5	1	20	81.65	106.2	1.22E-02	6.96E-02	1.00E+00
GO:0070813	hydrogen sulfide metabolic process	5	1	20	81.65	106.2	1.22E-02	6.96E-02	1.00E+00
GO:1990748	cellular detoxification	4	1	25	102.06	141.61	9.76E-03	6.77E-02	1.00E+00
GO:0006108	malate metabolic process	4	1	25	102.06	141.61	9.76E-03	6.77E-02	1.00E+00
GO:0019666	nitrogenous compound fermentation	4	1	25	102.06	141.61	9.76E-03	6.77E-02	1.00E+00
GO:0019660	glycolytic fermentation	4	1	25	102.06	141.61	9.76E-03	6.77E-02	1.00E+00
GO:0019673	GDP-mannose metabolic process	3	1	33.3	136.08	212.43	7.33E-03	5.71E-02	1.00E+00
GO:0046033	AMP metabolic process	3	1	33.3	136.08	212.43	7.33E-03	5.71E-02	1.00E+00
GO:0009298	GDP-mannose biosynthetic process	3	1	33.3	136.08	212.43	7.33E-03	5.71E-02	1.00E+00
GO:0010212	response to ionizing radiation	3	1	33.3	136.08	212.43	7.33E-03	5.71E-02	1.00E+00
GO:0019655	glycolytic fermentation to ethanol	3	1	33.3	136.08	212.43	7.33E-03	5.71E-02	1.00E+00
GO:0043458	ethanol biosynthetic process involved in glucose fermentation to ethanol	3	1	33.3	136.08	212.43	7.33E-03	5.71E-02	1.00E+00
GO:0006167	AMP biosynthetic process	3	1	33.3	136.08	212.43	7.33E-03	5.71E-02	1.00E+00
GO:0046292	formaldehyde metabolic process	2	1	50	204.12	424.91	4.89E-03	5.24E-02	1.00E+00
GO:0000947	amino acid catabolic process to alcohol via Ehrlich pathway	2	1	50	204.12	424.91	4.89E-03	5.24E-02	1.00E+00
GO:0034310	primary alcohol catabolic process	2	1	50	204.12	424.91	4.89E-03	5.24E-02	1.00E+00
GO:0110095	cellular detoxification of aldehyde	2	1	50	204.12	424.91	4.89E-03	5.24E-02	1.00E+00
GO:0000955	amino acid catabolic process via Ehrlich pathway	2	1	50	204.12	424.91	4.89E-03	5.24E-02	1.00E+00
GO:0006068	ethanol catabolic process	2	1	50	204.12	424.91	4.89E-03	5.24E-02	1.00E+00
GO:0044208	'de novo' AMP biosynthetic process	2	1	50	204.12	424.91	4.89E-03	5.24E-02	1.00E+00
GO:0033859	furaldehyde metabolic process	2	1	50	204.12	424.91	4.89E-03	5.24E-02	1.00E+00
GO:0110096	cellular response to aldehyde	2	1	50	204.12	424.91	4.89E-03	5.24E-02	1.00E+00
GO:0046294	formaldehyde catabolic process	2	1	50	204.12	424.91	4.89E-03	5.24E-02	1.00E+00
GO:1900695	regulation of N',N'',N'''-triacetylfusarinine C biosynthetic process	1	1	100	408.25	inf	2.45E-03	5.24E-02	6.30E-01
GO:0006069	ethanol oxidation	1	1	100	408.25	inf	2.45E-03	5.24E-02	6.30E-01

Supplementary Table ST4. Differentially abundant proteins in SMC-exposed IMV 00236-30

Accession	EggNog accession no.	EggNog annotated function / activity	IMV 00236-30/IMV 00236 log2 FC	t-test
BS090_000939	ENOG410PFHR	isocitrate dehydrogenase NADP	1.03	1.87E-02
BS090_007434	ENOG410PI78	phosphoenolpyruvate carboxykinase	1.04	1.25E-03
BS090_000071	ENOG410PFIA	Component of the ubiquinol-cytochrome c reductase complex	1.16	1.95E-03
BS090_001881	ENOG410PGND	Isocitrate lyase	1.23	8.71E-03
BS090_001715	ENOG410PGTG	NADH flavin oxidoreductase NADH oxidase family protein	1.25	3.00E-03
BS090_001053	ENOG410PGVS	oxidoreductase	1.45	2.24E-03
BS090_002404	ENOG41KOG2964	agmatinase	1.01	6.39E-03
BS090_002067	ENOG410QDDF	Aminotransferase family protein (LolT)	1.12	4.72E-03
BS090_005078	ENOG410PGVJ	Asparaginase	1.56	7.63E-03
BS090_011896	ENOG410PK51	Glyco_18	1.21	9.04E-03
BS090_000502	ENOG410PJKF	Catalyzes the epimerization of the S- and R-forms of NAD(P)HX	1.28	7.07E-03
BS090_000044	ENOG410PMJX	chitin deacetylase-like mannoprotein MP98	1.58	8.22E-03
BS090_001404	ENOG41KOG1458	Fructose-1,6-bisphosphatase	1.67	6.92E-03
BS090_003824	ENOG410PMRY	LysM domain	1.93	9.34E-03
BS090_000013	ENOG410PMJX	chitin deacetylase-like mannoprotein MP98	1.95	1.89E-02
BS090_001871	ENOG410PG7M	Exo-polygalacturonase	2.14	3.38E-03
BS090_010953	ENOG410PMF7	Chitin recognition protein	2.31	8.94E-04
BS090_003576	ENOG410PF94	Involved in biosynthesis of the thiamine precursor thiazole	1.77	7.86E-03
BS090_002486	ENOG410PFV1	Patched sphingolipid transporter (Ncr1)	1.14	1.07E-03
BS090_001853	ENOG410PG8Y	Ribosomal protein L15	1.13	1.89E-03
BS090_000406	ENOG410PN3M	40S ribosomal protein S12	1.44	2.67E-03
BS090_000082	ENOG41KOG1577	)-reductase	1.13	2.05E-03
BS090_010922	ENOG410PX4S	OsmC-like protein	1.12	3.34E-03
BS090_002416	ENOG41KOG1339	aspartic	1.42	3.68E-03
BS090_010341	ENOG410PJAB	thioredoxin reductase	1.55	5.80E-03
BS090_008934	ENOG410PFME	Thiamine biosynthesis protein (Nmt1)	1.30	5.87E-05
BS090_000547	ENOG410PFME	Thiamine biosynthesis protein (Nmt1)	1.84	1.90E-03
BS090_011103	ENOG410PPEB	dehydrogenase reductase family	1.06	1.43E-02
BS090_009219	ENOG410PHHS	Iron transport multicopper oxidase	1.14	2.98E-02
BS090_002395	ENOG410PKZK	oxidoreductase	1.50	5.60E-04
BS090_009534	ENOG410PJVI	nitroreductase	1.02	1.88E-04
BS090_004854	ENOG410PS59	Protein of unknown function (DUF3632)	1.10	6.55E-03
BS090_011127	ENOG410PFIQ	1,3-beta-glucanosyltransferase gel2	1.16	9.59E-04
BS090_002843	ENOG410PMUU	NA	1.20	4.46E-03
BS090_001072	ENOG410IGIF	AHNAK nucleoprotein	1.37	6.08E-03
BS090_004853	ENOG410Q2K1	ribonuclease	1.38	1.82E-02
BS090_002049	ENOG410PH8E	acid phosphatase	1.82	2.56E-03
BS090_006117	ENOG410PP1Q	Necrosis inducing protein (NPP1)	1.89	4.16E-03
BS090_000947	ENOG410XZM9	NA	2.30	7.43E-04
BS090_002760	ENOG41COG5048	Zinc finger protein	2.38	4.46E-03
BS090_002839	ENOG410PQZY	cell wall protein	2.96	8.08E-03
BS090_010530	ENOG410PHEY	Carboxylesterase family	1.02	8.05E-03
BS090_001068	ENOG410PIB8	transport from the ER, via the Golgi up to the trans	1.61	1.18E-02
BS090_001694	ENOG41KOG3135	Minor allergen Alt a	1.06	4.81E-03
BS090_001930	ENOG4106SRQ	#N/A	1.02	1.50E-02

BS090_006202	ENOG4113GX0	#N/A	1.07	1.84E-02
BS090_007239	ENOG410VI07	#N/A	1.14	1.14E-02
BS090_002215	ENOG4105FPR	#N/A	1.32	1.24E-03
BS090_002853	ENOG411DU5B	#N/A	1.40	3.00E-03
BS090_001291	0	#N/A	1.43	1.11E-02
BS090_001915	ENOG41arCOG07840	#N/A	1.44	9.78E-04
BS090_004925	ENOG410340Q	#N/A	1.57	1.70E-03
BS090_002860	0	#N/A	1.58	3.72E-03
BS090_006060	ENOG410UGBW	#N/A	1.61	2.77E-02
BS090_001703	0	#N/A	1.71	9.25E-03
BS090_001721	ENOG4105D4U	#N/A	1.84	1.88E-03
BS090_006728	ENOG410PIVD	Nuclear and cytoplasmic polyadenylated RNA-binding protein pub1	-2.64	4.86E-03
BS090_011566	ENOG410PGW8	splicing factor 3b	-1.88	1.34E-03
BS090_003449	ENOG410PGB0	Pre-mRNA-processing protein 45	-1.82	7.01E-03
BS090_002280	ENOG410PHXQ	ribonuclease T2	-1.70	4.97E-04
BS090_005913	ENOG410PH4U	ATP-dependent RNA helicase involved in 40S ribosomal subunit biogenesis	-1.62	1.70E-02
BS090_004639	ENOG410PG74	PAB1 binding protein	-1.29	2.57E-02
BS090_005026	ENOG410PI0I	RNA splicing factor Pad-1	-1.01	1.15E-02
BS090_005182	ENOG410PFFE	SIR2 family histone deacetylase	-2.59	1.02E-02
BS090_011426	ENOG410PRZF	Histone H1	-2.14	1.73E-03
BS090_009256	ENOG410PMG6	HMG box protein	-1.40	1.62E-02
BS090_005540	ENOG410PFTT	Paired amphipathic helix protein	-1.23	1.72E-02
BS090_010931	ENOG410PFJS	complex subunit	-1.08	1.09E-02
BS090_011296	ENOG410PNTK	Component of nucleosome which plays a central role in DNA double strand break (DSB) repair	-1.04	1.48E-02
BS090_005644	ENOG41KOG1758	atp synthase	-2.40	4.70E-03
BS090_008509	ENOG410PS16	Mitochondrial ATP synthase epsilon chain domain-containing protein	-2.04	2.62E-03
BS090_008223	ENOG410PNQY	Iron sulfur cluster assembly protein	-1.59	8.26E-03
BS090_011473	ENOG410PJA9	Vacuolar ATP synthase subunit e	-1.53	5.63E-03
BS090_004112	ENOG410PUCE	nitrate reductase	-1.51	1.16E-02
BS090_006999	ENOG410PFI6	Electron transfer flavoprotein	-1.33	2.00E-03
BS090_005666	ENOG410PH2F	Mitochondrial membrane ATP synthase (F(1)F(0) ATP synthase or Complex V)	-1.28	4.59E-03
BS090_003274	ENOG410PFBB	Stomatin family	-1.27	8.98E-05
BS090_011347	ENOG410PNPT	Cytochrome c oxidase polypeptide VIa	-1.26	1.29E-03
BS090_010935	ENOG410PFFW	Electron transfer flavoprotein	-1.23	1.25E-02
BS090_003155	ENOG410PNBT	Regulatory protein SUAPRGA1	-1.19	5.65E-03
BS090_011745	ENOG410PNH4	conserved hypothetical protein	-1.14	4.04E-02
BS090_004086	ENOG410PFM5	inorganic pyrophosphatase	-1.11	4.06E-02
BS090_000792	ENOG410PN6K	Mitochondrial membrane ATP synthase (F(1)F(0) ATP synthase or Complex V)	-1.02	1.27E-02
BS090_009724	ENOG410PR43	Cell division control protein	-2.53	3.65E-05
BS090_003607	ENOG410PJ75	Striatin Pro11	-1.49	2.80E-02
BS090_011383	ENOG410PS00	dsDNA-binding protein PDCD5	-1.19	2.87E-02
BS090_007016	ENOG410PG21	Carboxypeptidase	-1.56	6.31E-03
BS090_010423	ENOG410PHYE	GYF domain protein	-1.34	3.21E-02
BS090_009469	ENOG410PGKB	Aminotransferase	-1.27	3.06E-03
BS090_003500	ENOG410PKAW	Carboxypeptidase S1	-1.26	2.04E-04
BS090_008672	ENOG410PFWG	Isocitrate dehydrogenase	-1.21	7.90E-03
BS090_004087	ENOG410QEDC	Phosphoglycerate mutase	-2.34	9.93E-05
BS090_004931	ENOG410PM9J	Major intrinsic protein	-2.02	3.61E-02
BS090_011280	ENOG410PKN9	Oxalate decarboxylase	-1.91	8.40E-03

BS090_011829	ENOG410PMDW	alpha-amylase	-1.66	1.43E-03
BS090_003291	ENOG410PM6H	Glucanase	-1.51	4.22E-03
BS090_008425	ENOG410PIQS	Snf1 kinase complex beta-subunit Gal83	-1.49	2.12E-03
BS090_003039	ENOG410PH6W	Cell Wall	-1.47	9.13E-04
BS090_002903	ENOG410PJIN	Glycolipid transfer protein HET-C2	-1.37	3.43E-02
BS090_006133	ENOG410PF9K	glyceraldehyde-3-phosphate dehydrogenase	-1.17	5.18E-03
BS090_008700	ENOG410PK8I	WSC domain	-1.16	5.40E-03
BS090_007695	ENOG410PJ6P	glucan 1,4-alpha-glucosidase	-1.13	2.36E-03
BS090_011008	ENOG410PG84	Beta-glucosidase	-1.10	5.27E-04
BS090_010859	ENOG410PGWP	mannose-6-phosphate isomerase	-1.09	2.80E-02
BS090_001701	ENOG410PR96	Lipid transfer protein	-1.66	1.32E-03
BS090_009740	ENOG410PFKZ	3-keto-acyl-CoA thiolase	-1.01	1.03E-02
BS090_007862	ENOG410PQSK	60S ribosomal protein L22	-2.52	1.56E-03
BS090_006228	ENOG410PP4P	L-PSP endoribonuclease family protein (Hmf1)	-2.13	5.69E-03
BS090_009967	ENOG410PRWG	60S acidic ribosomal protein P1	-1.81	1.19E-02
BS090_006330	ENOG410PQ50	ribosome biogenesis protein Nhp2	-1.65	3.30E-03
BS090_001464	ENOG410PRUB	60S acidic ribosomal protein P2	-1.53	1.78E-03
BS090_000480	ENOG410PFEB	seryl-trna synthetase	-1.48	1.36E-02
BS090_003537	ENOG410PI34	Component of the eukaryotic translation initiation factor 3 (eIF-3) complex	-1.35	7.24E-03
BS090_002466	ENOG410PQS0	Required for the processing of the 20S rRNA-precursor to mature 18S rRNA in a late step of the maturation of 40S ribosomal subunits	-1.35	3.65E-03
BS090_006863	ENOG410PPAS	60s ribosomal protein	-1.35	2.19E-04
BS090_005915	ENOG410PHV2	prolyl-tRNA synthetase	-1.24	6.15E-03
BS090_002173	ENOG410PP8V	60S ribosomal protein L31	-1.23	7.84E-03
BS090_009099	ENOG410PGC2	Eukaryotic translation initiation factor 5	-1.18	3.88E-02
BS090_011340	ENOG410PNPS	Translation initiation factor	-1.16	5.27E-03
BS090_000015	ENOG410PMV6	40s ribosomal protein S17	-1.12	3.60E-03
BS090_003273	ENOG410PN86	La domain	-1.03	1.19E-02
BS090_010416	ENOG410PP4I	Component of the Mediator complex	-1.85	5.60E-03
BS090_007173	ENOG410PRUP	DNA-binding protein that induces severe bending of DNA	-1.82	3.03E-03
BS090_009682	ENOG41KOG1474	bromodomain containing	-1.77	1.06E-02
BS090_011032	ENOG410PK35	urease accessory protein UreG	-1.71	4.71E-03
BS090_000830	ENOG41KOG3598	Mediator complex, subunit	-1.58	1.98E-02
BS090_007807	ENOG410PPVK	Component of the nascent polypeptide-associated complex (NAC)	-1.44	4.78E-03
BS090_009793	ENOG410PQRP	DNA-directed RNA	-1.41	1.77E-02
BS090_004360	ENOG41101AM	megakaryoblastic leukemia (translocation) 1	-1.30	2.72E-03
BS090_002005	ENOG410PRUP	DNA-binding protein that induces severe bending of DNA	-1.04	2.61E-03
BS090_010694	ENOG41KOG0011	repair protein	-1.77	1.56E-04
BS090_009515	ENOG410PFS9	Multifunctional regulator of mitochondrial architecture and protein biogenesis	-1.44	1.80E-03
BS090_008141	ENOG410PRTR	Heat shock protein	-3.13	6.96E-03
BS090_009852	ENOG410PSDM	Glutaredoxin	-2.47	6.17E-03
BS090_008183	ENOG410PH8I	Secreted aspartic endopeptidase	-2.19	1.69E-03
BS090_010009	ENOG410PQV2	prefoldin subunit 6	-2.07	3.52E-02
BS090_010805	ENOG410PMR5	component of the PAM complex	-2.03	1.03E-02
BS090_005718	ENOG410PGPE	Disulfide-isomerase	-1.90	1.72E-04
BS090_008147	ENOG41KOG0541	peroxiredoxin	-1.81	3.06E-03
BS090_009384	ENOG410PI5I	Tripeptidyl-peptidase	-1.63	1.01E-03
BS090_007304	ENOG410PHFF	Protease S8 tripeptidyl peptidase I	-1.63	2.98E-03
BS090_004399	ENOG410PPJH	Heat shock protein	-1.59	1.87E-03

BS090_009030	ENOG410PP19	Peptidyl-prolyl cis-trans isomerase	-1.34	1.91E-03
BS090_005149	ENOG410PP3T	Subunit 3	-1.33	2.63E-03
BS090_011316	ENOG410PKHZ	protein-L-isoaspartate O-methyltransferase	-1.32	7.31E-03
BS090_010972	ENOG410PP80	Cupin domain protein	-1.28	3.67E-03
BS090_010452	ENOG410PQY3	Peptidyl-prolyl cis-trans isomerase	-1.24	1.83E-02
BS090_005834	ENOG410PJ50	26S proteasome non-ATPase regulatory subunit 11	-1.20	6.64E-04
BS090_008028	ENOG410PPYQ	ubiquitin conjugating enzyme	-1.12	4.88E-02
BS090_011023	ENOG410PNQ9	Peptidyl prolyl cis-trans isomerase Cyclophilin	-1.01	1.52E-03
BS090_002516	ENOG41KOG3574	solute carrier family 33 (acetyl-CoA transporter)	-2.50	9.01E-03
BS090_009331	ENOG410PPPY	Rhodanese domain protein	-2.28	2.11E-03
BS090_010245	ENOG410PN4F	Destroys radicals which are normally produced within the cells and which are toxic to biological systems	-1.26	1.23E-02
BS090_007815	ENOG410PKP5	Zinc-binding dehydrogenase	-1.16	1.84E-04
BS090_007164	ENOG410PR6N	LEA domain protein	-3.24	2.42E-03
BS090_006317	ENOG410PQM1	NA	-3.16	5.55E-04
BS090_005752	ENOG410PZN1	Conserved serine-rich protein	-2.69	6.35E-03
BS090_006452	ENOG410Q2VF	NA	-2.61	1.09E-03
BS090_008946	ENOG410Q2B6	NA	-2.60	4.34E-04
BS090_006001	ENOG410PSXE	conidiation protein Con-6	-2.54	7.89E-03
BS090_003732	ENOG410PNH7	GPI-anchored cell wall organization protein Ecm33	-2.53	2.22E-03
BS090_004763	ENOG410PSXE	conidiation protein Con-6	-2.53	2.06E-03
BS090_000529	ENOG410PST5	NA	-2.49	3.48E-02
BS090_007322	ENOG410PRJB	RNA binding protein	-2.46	1.29E-03
BS090_009941	ENOG410PS54	NA	-2.45	1.26E-03
BS090_006154	ENOG410PU0Y	NA	-2.43	3.17E-03
BS090_010760	ENOG410PSSD	Mitochondrial ATPase inhibitor, IATP	-2.31	1.67E-03
BS090_004636	ENOG410Q2RZ	NA	-2.31	4.65E-04
BS090_000112	ENOG410PRFU	RPEL repeat protein	-2.30	1.90E-02
BS090_003527	ENOG410QE02	NA	-2.29	3.19E-03
BS090_004479	ENOG410Q2B7	NA	-2.29	4.05E-04
BS090_007128	ENOG410PRV4	Seed maturation protein	-2.27	6.70E-03
BS090_008602	ENOG410PTHS	Protein of unknown function (DUF2611)	-2.24	1.02E-03
BS090_011952	ENOG410PI6I	conserved hypothetical protein	-2.21	5.37E-03
BS090_006176	ENOG410Q1IQ	NA	-2.17	3.17E-03
BS090_005118	ENOG410PSQJ	conidiation-specific protein	-2.10	1.62E-03
BS090_005412	ENOG410PXJZ	Inherit from NOG: conserved hypothetical protein	-2.09	1.64E-02
BS090_008779	ENOG410PNSC	Cystathionine beta-synthase	-2.06	2.79E-02
BS090_005559	ENOG410PKGM	BAR domain protein	-1.95	6.07E-05
BS090_005352	ENOG410Q2H5	Solid-state culture expressed protein (Aos23)	-1.95	4.52E-03
BS090_006318	ENOG410PTME	Inherit from NOG: Conserved hypothetical, protein	-1.94	7.14E-03
BS090_010560	ENOG410JDIQ	kinetoplast-associated protein-like protein	-1.88	5.77E-03
BS090_002110	ENOG410PQB3	conserved hypothetical protein	-1.88	9.18E-03
BS090_011465	ENOG410PQWT	conserved hypothetical protein	-1.84	2.30E-03
BS090_005992	ENOG410PJPQ	Polysaccharide Lyase family 7	-1.84	3.13E-03
BS090_010520	ENOG410PGA2	nuclear segregation protein	-1.80	3.85E-03
BS090_010137	ENOG410Q28G	NA	-1.75	5.74E-03
BS090_007062	ENOG410PSB5	tubulin-specific chaperone Rbl2	-1.75	1.20E-03
BS090_007758	ENOG410PPQW	Phosphatidylethanolamine-binding protein	-1.73	4.89E-03
BS090_009081	ENOG410PN9M	NA	-1.73	8.37E-03
BS090_003892	ENOG410PSQJ	conidiation-specific protein	-1.71	5.15E-03
BS090_011054	ENOG410PPXV	protein FMP21, mitochondrial	-1.68	5.73E-03
BS090_011794	ENOG410PHGI	sister chromatid separation protein	-1.67	1.95E-02

BS090_004466	ENOG410PSTV	NA	-1.61	2.33E-03
BS090_011070	ENOG410Q1UP	NA	-1.59	8.24E-03
BS090_007093	ENOG410PTYF	NA	-1.56	2.52E-03
BS090_004532	ENOG410PKIC	C2H2 finger domain protein	-1.56	2.72E-02
BS090_011638	ENOG410Q252	Lipase (class 2)	-1.54	2.50E-04
BS090_008428	ENOG410PI67	Ubiquinone biosynthesis protein	-1.49	3.09E-02
BS090_007213	ENOG410PKGM	BAR domain protein	-1.47	5.02E-03
BS090_011146	ENOG410PTF1	Oligosaccaryltransferase	-1.45	2.33E-02
BS090_010337	ENOG410PFK7	1,3-beta-glucanosyltransferase	-1.43	1.84E-02
BS090_005528	ENOG410PRYB	NA	-1.41	5.06E-04
BS090_005230	ENOG410PI5Y	conserved hypothetical protein	-1.39	7.70E-04
BS090_011025	ENOG410PN4Q	peroxisomal membrane anchor protein	-1.38	1.39E-04
BS090_000871	ENOG410IGIF	AHNAK nucleoprotein	-1.35	5.74E-04
BS090_011435	ENOG410PPBT	conserved hypothetical protein	-1.35	2.13E-04
BS090_010639	ENOG410PRAU	NA	-1.33	1.23E-02
BS090_007679	ENOG410PIQQ	conserved hypothetical protein	-1.29	1.61E-03
BS090_007997	ENOG410PPXQ	Telomere and ribosome associated protein Stm1	-1.25	9.49E-04
BS090_000617	ENOG410Q2VG	NA	-1.19	7.58E-03
BS090_007753	ENOG410PJJ6	conserved hypothetical protein	-1.19	4.79E-03
BS090_010469	ENOG410PRRT	Allergen Asp	-1.19	5.61E-03
BS090_008781	ENOG410PS0S	Protein of unknown function (DUF3602)	-1.18	1.91E-03
BS090_005942	ENOG410PQ8Q	Anticodon-binding domain	-1.17	1.51E-02
BS090_011387	ENOG410PQWI	conserved hypothetical protein	-1.15	2.52E-02
BS090_002170	ENOG410PQ08	SCP-2 sterol transfer family	-1.12	1.79E-04
BS090_000699	ENOG410PQP4	NA	-1.11	3.18E-03
BS090_011853	ENOG410PZDS	NA	-1.10	1.39E-02
BS090_000811	ENOG410PIST	LEA domain protein	-1.10	1.36E-02
BS090_005243	ENOG410PHK1	DUF431 domain-containing protein	-1.09	1.45E-03
BS090_009966	ENOG410PFAN	conserved hypothetical protein	-1.08	1.03E-02
BS090_009670	ENOG410PPAG	conserved hypothetical protein	-1.08	4.43E-03
BS090_003171	ENOG410PHFS	conserved hypothetical protein	-1.07	3.08E-03
BS090_009832	ENOG410PNG4	conserved hypothetical protein	-1.07	6.59E-04
BS090_000725	ENOG410PTWP	NA	-1.06	5.21E-04
BS090_002632	ENOG410PTGR	NA	-1.06	7.91E-03
BS090_011265	ENOG410PP64	Nadh-ubiquinone oxidoreductase	-1.04	4.14E-03
BS090_007300	ENOG410PJGK	Alpha beta hydrolase	-1.04	3.40E-03
BS090_011732	ENOG410Q1RQ	NA	-1.04	7.17E-03
BS090_010144	ENOG410PT5N	NA	-1.03	1.07E-02
BS090_008282	ENOG410PTR0	NA	-1.01	2.75E-02
BS090_005471	ENOG410PPTK	conserved hypothetical protein	-1.00	2.09E-02
BS090_003488	ENOG410PKN8	Carboxylesterase family	-2.03	1.63E-02
BS090_003024	ENOG410PIQP	PH domain protein	-1.58	3.26E-03
BS090_003004	ENOG410PIUR	NTF2 and RRM domain protein	-1.20	1.00E-02
BS090_005758	ENOG410PGRU	kinase, PRP4	-1.06	2.34E-02
BS090_004880	ENOG410PGW3	CAMP-dependent protein kinase regulatory subunit	-1.01	2.57E-03
BS090_010475	ENOG410PH63	Oxysterol binding protein	-1.00	1.44E-03
BS090_000917	ENOG410PRVI	mitochondrial import receptor subunit tom22	-2.29	5.21E-03
BS090_011191	ENOG410PK1B	SNARE domain protein	-1.68	6.27E-04
BS090_009233	ENOG410PRXA	protein transport protein sec61	-1.48	1.96E-02
BS090_009608	ENOG410PI2Z	vacuolar protein sorting-associated protein vps5	-1.46	3.21E-03
BS090_006025	ENOG410PF9U	K17065 dynamin 1-like protein EC 3.6.5.5	-1.43	1.01E-02
BS090_005792	ENOG410PFAY	peroxisomal membrane anchor protein	-1.37	8.28E-03
BS090_003720	ENOG410PNHZ	Integral ER membrane protein	-1.11	3.57E-03

BS090_004992	ENOG410PN4Z	Component of the nascent polypeptide-associated complex (NAC)	-1.10	8.56E-03
BS090_009215	ENOG410PH90	BAP31 domain protein	-1.44	1.52E-02
BS090_006534	ENOG410PMCJ	3-beta hydroxysteroid dehydrogenase/isomerase family	-1.29	4.11E-03
BS090_010717	ENOG410QE97	Dual specificity phosphatase	-1.01	1.14E-03
BS090_006448	ENOG410PKM7	chromatin remodeling complex subunit (Arp8)	-1.81	3.88E-02
BS090_004144	ENOG410PNPX	Tropomyosin	-1.58	4.97E-03
BS090_007297	ENOG410PIBY	F-actin-capping protein	-1.44	1.13E-02
BS090_001642	ENOG410PNPX	Tropomyosin	-1.26	1.64E-02
BS090_008504	ENOG41KOG1735	actin-depolymerizing factor	-1.18	7.34E-03
BS090_000535	ENOG41KOG0046	lymphocyte cytosolic protein 1 (L-plastin)	-1.10	5.60E-04
BS090_009365	ENOG410PQR4	Binds to actin and affects the structure of the cytoskeleton	-1.06	1.92E-02
BS090_004260	#N/A	#N/A	-2.58	4.92E-03
BS090_006661	#N/A	#N/A	-2.37	4.17E-03
BS090_006040	ENOG4112TDX	#N/A	-2.22	6.92E-03
BS090_006666	ENOG410XI5N	#N/A	-2.04	6.90E-04
BS090_004822	#N/A	#N/A	-2.03	1.39E-02
BS090_012017	#N/A	#N/A	-1.85	2.33E-03
BS090_008231	ENOG410V0NJ	#N/A	-1.67	7.46E-03
BS090_005624	#N/A	#N/A	-1.61	5.14E-03
BS090_006775	ENOG411E6PQ	#N/A	-1.61	4.56E-03
BS090_006668	0	#N/A	-1.58	9.65E-03
BS090_005819	ENOG4112QCH	#N/A	-1.56	3.21E-03
BS090_011138	0	#N/A	-1.33	1.57E-02
BS090_005711	ENOG4113M17	#N/A	-1.28	1.16E-02
BS090_008573	ENOG410X7ZR	#N/A	-1.23	2.70E-03
BS090_000479	#N/A	#N/A	-1.20	3.85E-02
BS090_006726	ENOG410VI9I	#N/A	-1.14	2.04E-03
BS090_011954	ENOG4114FV5	#N/A	-1.05	1.90E-02

* Log2 fold change of ISSFT-021-30 min compared to unexposed ISSFT-021 counterpart ($P < 0.05$)
